# Phytochemicals and bioactive functional ingredients from *rosa damascena*: from extraction to application in the food and healthcare sectors

**DOI:** 10.1016/j.fochx.2025.103202

**Published:** 2025-10-25

**Authors:** Arezou Khezerlou, Keyhan Mohammadi, Amirhossein Abedini, Maryam Alizadeh Sani, Mahmood Alizadeh Sani, David Julian McClements

**Affiliations:** a Nutrition Research Center, Tabriz University of Medical Sciences, Tabriz, Iran; b Department of Clinical Pharmacy, Faculty of Pharmacy, Tehran University of Medical Sciences, Tehran, Iran; c Student Research Committee, Department of Food Science and Technology, Faculty of Nutrition Science and Food Technology, National Nutrition and Food Technology Research Institute, Shahid Beheshti University of Medical Sciences, Tehran, Iran; d Department of Biology, Hakim Sabzevari University, Sabzevar, Iran; e Department of Food Science and Technology, School of Nutritional Sciences and Dietetics, Tehran University of Medical Sciences, Tehran, Iran; f Nutraceutics Research Center, Tehran University of Medical Sciences, Tehran, Iran; g Department of Food Science, University of Massachusetts Amherst, Amherst, MA 01003, USA

**Keywords:** Food formulation, Green extraction, Natural preservatives, Pharmaceutical applications, *Rosa damascena*, Techno-functional properties

## Abstract

*Rosa damascena* is a rich source of bioactive ingredients including flavonoids, anthocyanins, and terpenoids (like citronellol (30–40 %) and geraniol (20–30 %)), which endow significant antioxidant (IC₅₀ values of 0.2–0.5 mg/mL) and antimicrobial activity (MIC values of 0.05–0.5 % against food-borne pathogens). Compared to traditional methods, modern extraction techniques significantly improve yield, efficiency, and preserve the chemical integrity of bioactive compounds, such as supercritical fluid extraction can achieve a high yield of 92 %, and solvent-free microwave-assisted extraction that reduces processing time by up to 90 % compared to conventional methods. Due to the volatility and susceptibility of rose bioactive ingredients to degradation by light, air, moisture, and high temperatures, encapsulation technologies can enhance their stability by 60–80 %. Accordingly, the free and encapsulated forms of *Rosa damascena* essential oils and extracts serve as effective natural preservatives and shelf-life extenders in various food products, as well as in biodegradable active and intelligent packaging, enabling real-time spoilage monitoring (as indicator) and sustained food quality with significantly prolonged shelf life. Moreover, these bioactive compounds hold considerable potential for integration into functional foods and nutraceuticals, addressing health concerns by promoting improved gastrointestinal comfort, mood stabilization, migraine relief, and enhanced endocrine function. These advancements position *Rosa damascena* as a versatile and sustainable bio-resource with remarkable development of innovative, health-promoting products in the food and nutraceutical industries.

## Highlights

• REOs compounds possess antioxidants, antimicrobial & therapeutic effects.
• Green techniques (ultrasound, supercritical) optimize yield, stability, sustainability & food safety compliance.
• REOs act as natural preservatives, extend shelf life, improve nutrition in clean-label foods vs synthetics.
• Address gaps with omics for synergies, trials, encapsulation & sustainable cultivation for innovative food systems.

## Graphical abstract

**Figure:**
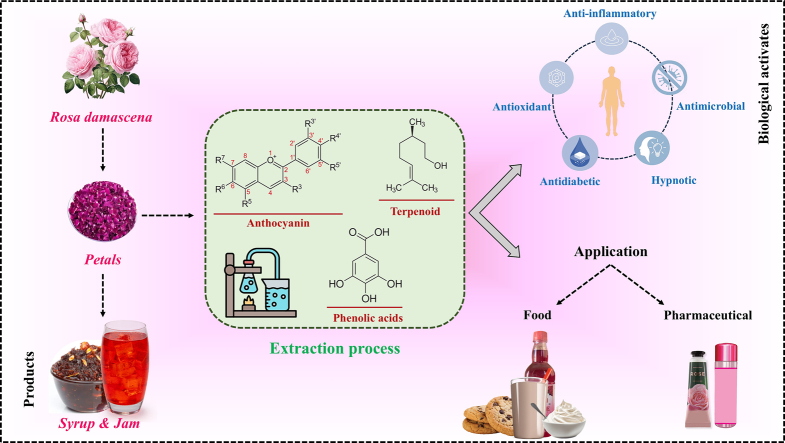

## 1 Introduction

Edible flowers have been widely used for nutrition and treatment of human diseases since the past until today (Pensamiento-Niño et al., 2024). *Rosa damascena* (*R. damascena*) is one of the most important edible flowers. This species flowers in different regions of the world, such as Iran, Bulgaria, India, Italy, Syria, France, Turkey, Russia, America, China, Korea, and Japan (Mileva et al., 2021). Historically, these flowers are known for their beautiful appearance and pleasant smells, but today, they are also used as a source of natural antioxidants, antimicrobials, aromas, pigments, and other functional ingredients (Baydar & Baydar, 2013; Dinçoğlu & Rugji, 2021). Based on their botanical features, more than 1000 species of rose flower have been identified and reported so far, but only a small fraction of them have been commercialized (Pereira et al., 2020). In some countries, especially Iran and Bulgaria, the cultivation and sale of roses and their derivatives, such as extracts and essential oils, is an old tradition (Mileva et al., 2021). Among these, rose oil is the most significant and is often referred to as “liquid gold. Bulgaria stands as the world's leading producer, accounting for over 80 % of global output (15–20 tons annually), primarily from the Kazanlak and Karlovo regions. Turkey is the second-largest producer, contributing approximately 15 % of the world's supply (Venkatesha et al., 2022). Other notable producers include India (200 kg of rose oil with cultivation 2500–3000 ha), Iran (166 kg), and Morocco, which collectively account for the remaining 5 % of global production (Pise et al., 2023).

**Fig. 1:**
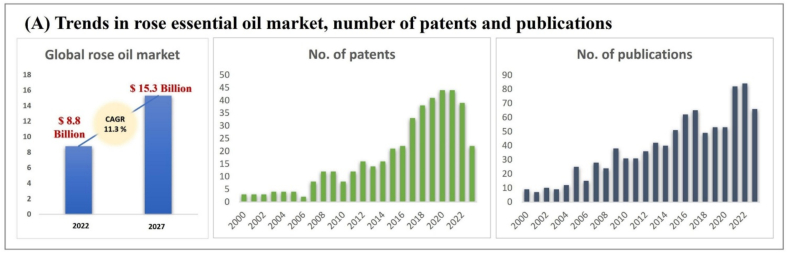
Predicted increase in global REO market (2022–2027); number of patents and research papers published on REOs. Reprinted with permission from (Anmol et al., 2025). Copyright © 2025 Elsevier.

Indeed, the application of rose essential oils (REOs) and their extracts have been of interest for many years (Fig. 1). Compositionally, REOs have been reported to contain a complex mixture of different bioactive phytochemicals (Ahadi et al., 2023). Rose oils have therefore been used for centuries in medicines, cosmetics, and foods for their desirable biological activities. Typically, extraction processes yield a rich array of bioactive phytochemicals, including flavonoids, terpenes, phenolic acids, and alcohols, each contributing uniquely to its overall functional properties (Guo et al., 2022). The main ingredients of REOs include limonene, linalool, ꞵ-phenethyl alcohol, cis-rose oxide, trans-rose oxide, citronellol, nerol, geraniol, eugenol, methyl eugenol, n-heptadecane, farnesol, nonadecene (C_19:1_), n-nonadecane (C_19_), n-eicosane (C_20_), and n-heneicosane (C_21_) (Mileva et al., 2021).

One of the most common applications of REOs and their extracts is as natural preservatives, as many of the phytochemicals it contains exhibit potent antioxidant and antimicrobial properties (Androutsopoulou et al., 2021; Chroho et al., 2022). The antioxidant constituents help to neutralize free radicals and protect lipids from oxidative damage, thereby allowing them to inhibit oxidation in foods and other commercial products, as well as in the human body (Mileva et al., 2021). Additionally, the antifungal and antibacterial constituents in REOs can protect foods and other products from contamination with spoilage or pathogenic microorganisms, thereby extending their shelf life and enhancing their safety (Liu et al., 2023; Mokhtari et al., 2023; Trendafilova et al., 2023). The strong preservative properties of REOs are particularly important as the food industry tries to replace synthetic additives with natural ones that are more sustainable and safer.

In pharmaceuticals and medicine, the therapeutic potential of REO is gaining attention for its application in treating various conditions. For instance, its anxiolytic properties help reduce stress and promote relaxation, thereby making it a popular choice in aromatherapy (Mahdood et al., 2022; Mokhtari et al., 2023). Furthermore, research indicates that rose oil can aid in skin healing, making it a valuable ingredient in skincare formulations designed to nourish and regenerate the skin (Akram et al., 2020; Mokhtari et al., 2023). Researchers have claimed a broad range of potential health benefits for extracts isolated from roses, including reducing depression, promoting mental relaxation, overcoming sexual disorders, muscle relaxation, fat-reduction, anti-ulcer, and anticancer effects (Imanieh et al., 2022; Kashani et al., 2016; Mokhtari et al., 2023).

The literature review reveals that *rosa damascena* is a rich source of bioactive phytochemicals, including flavonoids (e.g., quercetin, kaempferol), essential oils (e.g., geraniol, citronellol), and phenolic compounds, which exhibit significant antiviral, antioxidant, anti-inflammatory, and antimicrobial properties. In food applications, *rosa damascena* extract enhances shelf-life and nutritional value as natural preservatives and functional ingredients. In healthcare, *rosa damascena* show potential as therapeutic agents for viral infections, oxidative stress-related diseases, and inflammation (Ahadi et al., 2023; Alizadeh & Fattahi, 2021; He & Putra, 2025).

Research gaps include limited studies on optimized extraction techniques for maximizing bioactive yield, insufficient in vivo data validating efficacy and safety, and a lack of comprehensive proteomic/transcriptomic analyses to elucidate molecular interactions. Additionally, synergistic effects of combining *rosa damascena* phytochemicals with other bioactives remain underexplored, as do standardized protocols for their incorporation into food and pharmaceutical products. These gaps highlight the need for further research to explain *rosa damascena*'s potential into practical applications (Shabbir et al. 2020, Kayahan et al., 2024). More importantly, the reported studies only examined the therapeutic and biological effects of rose compounds, and the comprehensive review article did not explore the use and role of these rose compounds in food applications.

Therefore, this study for the first time aimed to critically review the phytochemical composition, extraction methods, encapsulation technologies, biological activities, and therapeutic effects of REOs and their extracts. In addition, the potential applications of REOs in food products/formulation, food packaging materials, pharmaceuticals, and health care products are also assessed. In fact, this review addresses the demand for natural bioactives in food and healthcare due to consumer preference for sustainable, safe alternatives. *Rosa damascena's* underutilized potential induces consolidating knowledge to guide its application in functional foods and therapeutics, addressing research gaps for practical innovation.

## 2 Phytochemical composition of REOs

Rose (*Rosa damascena*), also known as Damask Rose (in English) or Gole Mohammadi (in Iran), belongs to the *Rosaceae* family, which is believed to have originated in Iran (Rasouli et al., 2018, Shabbir et al. 2020). The phytochemical composition of REO depends on the genotype, cultivation conditions, climatic conditions, time and stage of flower harvesting, and the method of extraction (Omidi et al., 2022). The main group of chemical components in rose flowers include phenolic acids (e.g.*,* chlorogenic, caffeic, gallic, and coumaric), flavonoids (e.g.*,* quercetin, kaempferol, rutin, and epicatechin), terpenoids (e.g.*,* β-citronellol, geraniol, and nerol), and anthocyanins (Table 1) (Nasery et al. 2016, Rasouli et al., 2018, Anmol et al., 2025).

**Table 1:** The compositions of Damask rose bioactive agents reported in various studies.⁎

**Bioactive compound**	**Country**	**Extraction condition**	**Phytochemical Content**⁎	**Main compounds**	**Ref.**
Phenolic compound	Kosovo	Ultrasonication, Natural deep eutectic solvents and ethanol	Range: from 624.45 to 668.91 mg GAE/g	–	(Koraqi et al., 2024)
	Turkey	30 ml of 5 % sodium bicarbonate and 60 mL of ethyl acetate	Range: from 4115 to 2196 mg GEA 100 g^−1^ (at different periods)	–	(Kayahan et al., 2024)
	Morocco	Ethanol/water (70:30, *v*/v)	Average: 20.07 mg GAE/g dm	Gallic acid	(Chroho et al., 2022)
	Iran	Ethanol (70 %)	Range: from 134.568 to 217.728 mg CE/g	Gallic acid	(Memariani et al. 2015)
	Turkey	Hot: Soxhlet extractor, methanol at 60 °C Cold: Ultra Turrax mixer, methanol for 1 min	Hot: 233.56 mg GAE/g Cold: 344.45 mg GAE/g	Gallic acid	(Baydar & Baydar, 2013)
Flavanols	Turkey	Hot: Soxhlet extractor, methanol at 60 °C Cold: Ultra Turrax mixer, methanol for 1 min	Hot: 28.71 mg CE/g Cold: 29.76 mg CE/g	–	(Baydar & Baydar, 2013)
	Saudi Arabia	Methanol)80 %) *n*-Butanol fraction Aqueous fraction	Average: 21.01 mg Qur equivalent/g Average: 34.46 mg Qur equivalent/g Average: 3.08 mg Qur equivalent/g	–	(S et al. S, 2013)
Flavonoids	Kosovo	Ultrasonication, Natural deep eutectic solvents and ethanol	Range: from 61.90 to 86.12 MgCE/g	–	(Koraqi et al., 2024)
	Iran	Ultrasonication at 25 °C for 1 h, methanol	Average: 48.9 mg/g DW	Kaempferol 3-O-glucoside Quercetin 3-O-glucoside Quercetin 7-(6”galloylglucoside)	(Ahadi et al., 2023)
	Iran	Ethanol (70 %)	Range: from 15.84 to 22.8 mg CE/g		(Memariani et al. 2015)
Anthocyanin	Turkey	LC-MS/MS system, water: citric acid solution (99:1)	Average: 515.79 mg C_3_G Eq./100 g dm	Cyanidin-3,5-diglucoside Cyanidin-3-diglucoside	(Torusdağ & Bakkalbaşı, 2022)
	Iran (petal)	Methanol/ HCL (99:1)	Average: 37.48 μmoL/g DW (Hot pink petals) 2.12 μmoL/g DW (white petals)	–	(Kiani et al., 2024)
	Iran (EO)	Ultrasonication at 25 °C for 1 h, methanol	Average: 37.0 mg/g DW Range: from 0.0 to 37.0 mg/g DW	–	(Ahadi et al., 2023)
	Iran (EO)	Ultrasonication 600 W, at 30 °C for 30 min, methanol 80 %	Range: from 0.69 to 3.67 C_3_G mg /g DW	–	(Alizadeh & Fattahi, 2021)

GAE: Gallic Acid Equivalent; CE: Catechin Equivalent; QE: Quercetin Equivalent; C_3_G: Cyanidin-3-Glucoside, LC-MS/MS: Liquid chromatography-tandem mass spectrometry; dm: dry matter; Qur: quercetin; DW: dry weight; C_3_G Eq: cyanidin-3-O-glucoside equivalent.

⁎ Rang and average: Phytochemical Content.

Recent research on Damask rose from Anhui Province (Chin) further confirms this complexity, identifying 76 volatile compounds across different processed products. Among these, 28 volatiles were detected as key aroma-active compounds, with phenethyl alcohol and α-terpineol significantly influencing organoleptic attributes (He et al., 2024). They also contain various fatty acids, organic acids, carbohydrates, vitamins (A, C, D, E, and B_3_), and minerals (iron, zinc, calcium, phosphorus, sodium, manganese, and potassium) (Akram et al., 2020). Damask rose seed oil is of great importance because of its richness in ω-3 fatty acids, such as α-linolenic acid (Shabbir et al. 2020). β-citronellol, nonadecane, eugenol, and geraniol are important active compounds in rose oil. Rose flavor is strongly influenced by the presence of β-ionone, β-damascone, and β-damascenone compounds that are formed by the degradation of carotenoids (Huang et al., 2009).

Rose is also a plant source of natural pigments, including anthocyanins (Alizadeh & Fattahi, 2021), such as cyanidin, pelargonidin, peonidin, and malvidin (Karami et al., 2012; Wan et al., 2019). Research into the flower colors of roses has revealed that four anthocyanins, specifically 3-glucosides and 3,5-diglucosides of cyanidin and peonidin, are present in the flowers of wild rose species, whereas pelargonidin 3-glucoside and pelargonidin 3,5-diglucoside are more prevalent in cultivated rose varieties (Wang et al., 2023). Twelve flavonoids and four anthocyanins have been reported in rose extracts (Karami et al., 2012). In a study conducted by Omidi et al. (2022), the genotypes of *R. damascena* exhibited significant variation in petal color, ranging from dark pink (G3/Tehran genotype) to pale pink (G9/Fars genotype) and white (G2/Isfahan and G26/East Azerbaijan genotypes). Nevertheless, most of them were either pink or pinkish in color, which can be attributed to the presence of anthocyanins in the petals, such as pelargonidin and cyanidin (Omidi et al., 2022).

The varying compositions of Damask rose bioactive agents reported in Table 1 result from a complex interplay of genetic, environmental, and harvest factors (Manouchehri et al., 2018). Genetically distinct cultivars inherently produce different profiles of volatile and non-volatile compounds. Furthermore, agronomic practices (e.g., propagation and growth conditions), environmental variables (e.g., temperature, light, and soil type), harvest timing, and post-harvest processing methods all critically determine the final REO's yield and compositional quality (Venkatesha et al., 2022). A comprehensive understanding of these interacting factors is essential for standardizing quality and guiding the selection of optimal genotypes and processing techniques for specific industrial applications.

## 3 Extraction of REOs

REOs can be extracted from roses using a variety of traditional and emerging approaches (He & Putra, 2025; Manouchehri et al., 2018). Traditional methods of extracting EOs have been discussed in detail in previous reports (Katekar et al., 2022; Manouchehri et al., 2018). For this reason, this section focuses on emerging methods, including ionic liquid (ILs), hydro-distillation (HD), solvent-free microwave-assisted extraction (SFME), ohmic-assisted hydro-distillation (OAHD), supercritical carbon dioxide (CO_2_), and pulsed electric field (PEF) methods (Cui et al., 2024; Manouchehri et al., 2018; Patrascu & Radoiu, 2016). A detailed comparison of the advantages and disadvantages of each method, focusing on yield, energy consumption, scalability, and processing time, is summarized in Table 2. One of the most important issues when developing a suitable method for the extraction of EOs and their extracts is to calculate the optimum mass of the sample and volume of the solvent required to obtain the maximum yield and purity (Anmol et al., 2025; Cui et al., 2024).

**Table 2:** Comparative summary of REOs extraction methods.

**Method**	**Yield**	**Energy Consumption**	**Processing Time**	**Scalability**	**Advantages**	**Shortcoming**
HD	Very low	High	Long ∼ 1.5–2 h	High	• Simple • Low cost • Eco-friendly	• Needed to large amounts of raw materials • Damage to volatile compounds
ILs	High	Low	Long	Low (large scale: ILs with low viscosity)	• High efficiency • High selectivity • Good renewability	• High viscosity • High production cost • Usage of additional external force
SFME	High	Low	Short (10 times shorter than HD)	Easily to scale up	• Higher purity • Low cost • Reducing the use of solvents	• Required optimization to prevent the damage of heat-sensitive ingredients
OAHD	High	Low	Short (6 times shorter than HD)	Limited (High initial capital investment required for the specialized equipment)	• High thermal efficiency • Better process control • Better extract quality	• Negative effects on phytochemicals due to electrochemical reactions from electrode contact • Limited to electroconductive samples
Supercritical CO_2_ extraction	High	Moderate to High	Short	High	• Lower extraction temperature • Non-toxic • Reduced solvent residues • Easily recyclable	• Initial cost of equipment • Energy required to maintain supercritical conditions
PEF	High	Low	Very short (mS/μS)	Easy to scale up	• Cost-efficacy • Less solvent consumption • High selectivity	• High cost of equipment

**HD method**: HD is a traditional, eco-friendly method for extracting REOs by heating rose petals in water, generating steam that volatilizes aromatic compounds. The steam carries these compounds, which are then condensed in a cooling system and separated into oil and water layers. Rose oil, being lighter, is collected from the top (Kara et al. 2017). Although effective in preserving key fragrance components (citronellol and geraniol), it is time-consuming and may degrade heat-sensitive compounds if not carefully controlled (Katekar et al., 2022; Rajabi-Moghadam et al., 2025). For instance, Katekar et al. (2022) reported the best extraction yield of 0.07 % using 0.25 kg sample, 0.75 L solvent, 300 W power, and 1.5 h extraction (Katekar et al., 2022).

**Fig. 2:**
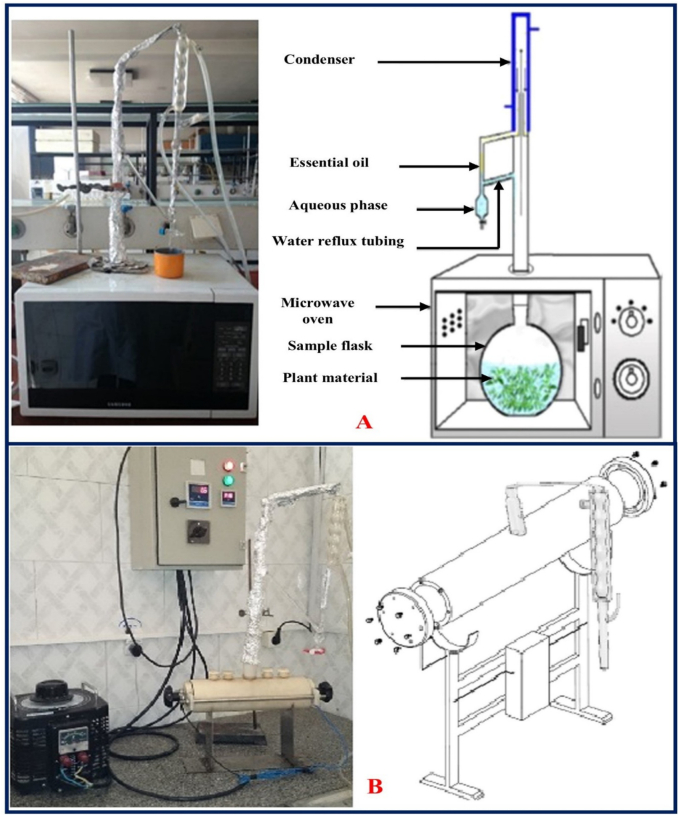
SFME **(A)**; and Ohmic heating **(B)** methods for extraction of REOs. Reprinted with permission from Ref. (Manouchehri et al., 2018). Copyright © 2018 Elsevier.

**SFME method**: The SFME method is used to extract REOs by treating rose samples with microwaves, as seen in Fig. 2A. Initially, the rose sample is placed in a Clevenger apparatus without solvent for 25 min. Then, sample is processed in a modified microwave oven at 1000 W for 3 min, followed by 400 W for 22 min. The extracted REOs are dried using sodium sulfate (Manouchehri et al., 2018). Studies report that SFME yields 0.02 % ± 0.0056 % (*w*/w), with oil accumulation in 0.29 ± 0.25 min and a total extraction time of 24.65 ± 0.031 min (Manouchehri et al., 2018).

**OAHD method:** The OAHD method is based on combining hydro-distillation with ohmic heating. The efficiency of this method depends on processing variables such as ohmic power level, frequency, temperature, and processing time (Farahnaky et al., 2010). For instance, Manouchehri et al. (2018) employed the OAHD method for the extraction of REOs from fresh rose and reported a yield, oil accumulation time, and total extraction time of 0.002 ± 0.047 %, 17.3 ± 0.33 min, and 42.33 ± 0.033 min, respectively (Manouchehri et al., 2018) (Fig. 2B).

**Supercritical CO**_**2**_ **extraction method**: The general principle of this method includes grinding up the plant material and then placing it in an appropriate extraction vessel. Then, CO_2_ gas is subjected to high temperatures and pressures to convert it into a supercritical fluid. A pump then forces the supercritical CO_2_ into the extraction vessel, where it penetrates into the plant material and extracts the EOs (Díaz-Reinoso et al., 2006). Cui et al. (2024) investigated the use of the supercritical CO_2_ method to extract EOs from roses and observed that the best extraction yield (8.99 %) was obtained using conditions of 10 % solvent (salt), 0.8 mm particle size, 10 L/h, 40 °C, and 35 MPa (Cui et al., 2024). Darvishi Nooshabadi et al.et al. (2024) demonstrated that supercritical fluid extraction of rose reached a high efficiency of 92 % under optimal conditions (180 bar, 45 °C, 180 min). Their economic evaluation estimated a fixed investment cost of $412,000 and a manufacturing cost of $220,000, with a capital return period of only 8 months, indicating strong industrial feasibility (Darvishi Nooshabadi et al., 2024).

**PEF method**: The PEF method is a non-thermal processing method, which uses high intensity electrical pulses to breakdown the structure of plant tissues, thereby facilitating extraction (Sani et al. 2024). Typically, the plant tissues are first positioned between two or more electrodes and then subjected to brief high-voltage electric field pulses that last from microseconds to milliseconds (Li et al., 2023). Yajun et al. (2017) investigated PEF for REO extraction and found that an electric field intensity of 20 kV/cm increased the yield by 50 % (Yajun et al., 2017).

**Ionic liquid method**: This method involves several key steps: the preparation of a suitable ionic liquid, mixing of the ionic liquid and rose sample, incubation at an elevated temperature, filtration, evaporation, and sample collection. As an example, rose is added to a quaternary ammonium salt with a cation-anion balance of 1:2 and incubated for 3 h at 75 °C (Shi et al., 2024). After the filtration of mixture, the ionic liquid is removed via a rotary evaporator at 55 °C to collect REOs. The yield is determined by comparing the mass of extracted REOs to the mass of the initial rose sample. Guo et al. (2022) evaluated twelve ionic liquids for extracting REOs, and found the optimal conditions to be a 1:4 mass ratio of rose sample to ionic liquid, 100 g of stripping agent, 4 h reflux time, and 75 °C extraction temperature (Guo et al., 2022).

**Fig. 3:**
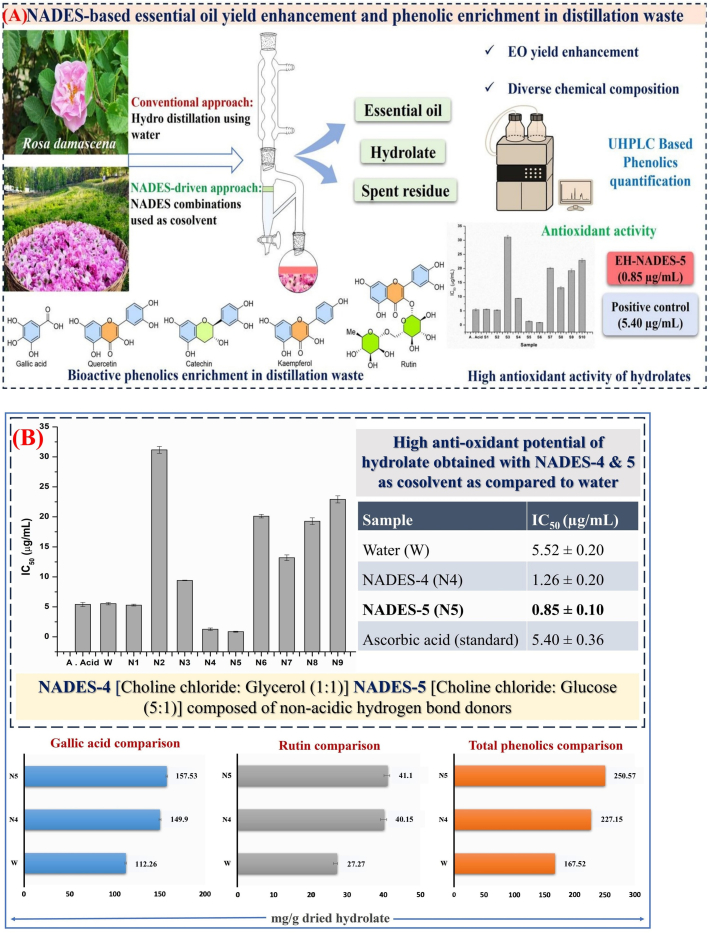
**A)** Schematic diagrams of REOs extraction using natural deep eutectic solvents (NADES) as co solvents in the conventional hydro distillation method. **B)** Antioxidant activity (IC_50_) and comparison of phenolics, Reprinted with permission from (Anmol et al., 2025). Copyright © 2025 Elsevier.

HD: Hydro-distillation; SFME: Solvent-free microwave-assisted extraction; OAHD: Ohmic-assisted hydro-distillation; PEF: pulsed electric field; ILs: Ionic liquid.

Natural deep eutectic solvents have been used as cosolvents with conventional hydro-distillation methods to enhance the extraction of REOs and other phytochemicals from rose petals (see Fig. 3A) (Anmol et al., 2025). This combined approach was shown increase the recovery, yield, and antioxidant capacity of the extracted essential oil (Fig. 3B).

Traditional extraction methods are widely adopted in industrial settings due to their high scalability and low initial costs. These methods are simple and effective for extracting EOs but require long processing times and large raw material inputs, resulting in lower yields (He & Putra, 2025). These methods achieve yields typically with max 0.069 % for REOs, meeting commercial benchmarks of solvent volume of 0.75 L at <0.25 kg. However, their prolonged processing times (1.5 h), power consumption (300 W), and thermal exposure result in loss of volatile compounds, compromising efficiency and quality (Katekar et al., 2022). In contrast, modern extraction methods, that mentioned above, offer superior yields (0.04–0.4 %) and significant energy savings with shorter extraction time (<15 min) (Villa et al. 2022). For instance, SFME achieves EOs yield in <3 min and OAHD in <18 min, significantly faster than HD (< 43 min), while SFME increase hydrocarbons and reduce monoterpene alcohols, a qualitative drawback for high-value applications (Manouchehri et al., 2018). Emerging methods (supercritical CO_2_ extraction and SFME) provide promising alternatives to traditional methods, delivering enhanced efficiency, selectivity, and environmental sustainability.

## 4 Encapsulation of REOs

Encapsulation technologies are commonly used to enhance the utilization and efficacy of rose extracts. For instance, REOs are predominantly hydrophobic substances with poor chemical stability and high volatility, which can make it challenging to incorporate them into aqueous based products and to ensure that these products have a sufficiently long shelf life. Mohammadi et al. (2021) encapsulated REOs in N-butyl palmitate/stearate particles that were relatively small (∼100 nm) (Mohammadi et al., 2021). Heydari et al. (2021) encapsulated REOs in biopolymer complexes fabricated from whey proteins and pectin. They observed that the best encapsulation efficiency was achieved at a ratio of 4:5 whey protein-to-pectin at pH 3, where there was a strong electrostatic attraction between the cationic whey proteins and anionic pectin molecules. They concluded that these biopolymer-based complexes could be used to successfully encapsulate rose oils for application in different food matrices (Kord Heydari et al., 2021). The release properties of REOs can be controlled using appropriate encapsulation technologies, which may be useful for certain applications. For instance, Xiao et al. (2019) investigated the release properties of REOs from particles made from maltodextrin and octenyl succinic anhydride starch. They showed that the phenols had the most delayed release and the esters had the fastest release. They found that the use of a 2:1 ratio of starch-to-maltodextrin provided the best conditions to obtain a relatively high REO loading capacity (45.3 ± 0.6 %). They concluded that the use of this formulation significantly increased the stability the release of the REOs. Moreover, increasing the temperature or relative humidity could be used to promote the release of the REOs from the biopolymer particles (Xiao et al., 2019). In another study, Khodadadi et al. (2024) studied the encapsulation of REOs by lignin nanoparticles. They showed that the optimal sample in terms of encapsulation efficiency (63.9 %) and physical properties was obtained when using a 2:1 ratio of REO-to-lignin nanoparticles. The REO encapsulated within the lignin nanoparticles had a significantly higher thermal stability and antioxidant activity than the control samples. In addition, the encapsulated group showed good antimicrobial properties against *S. aureus* and *E. coli*. The authors therefore concluded that lignin nanoparticles could be a potential delivery system for developing rose oil-based antimicrobial and antioxidant systems (Khodadadi et al., 2024).

Contri et al. (2016) investigated the potential of encapsulating REO in chitosan-based nanoparticles. They found that encapsulated REOs could effectively absorb ultraviolet light and inhibit lipid oxidation, thereby making them suitable for skin and cosmetic applications, where UV blocking and antioxidant properties are required (Contri et al., 2016). Qiu et al. (2022) investigated the use of apricot peel pectin and mung bean protein for rose oil microencapsulation. The highest encapsulation efficiency (89.9 %) was obtained using a 4:1 ratio of protein-to-pectin. These samples had good antioxidant properties, a stable release profile, and higher thermal stability than the control samples (Qiu et al., 2022). The same research team investigated the use of sodium alginate and perilla protein for the encapsulation of REO. They observed that the optimal ratio used for protein and sodium alginate was 6:1, which led to an encapsulation efficiency of 89.8 %. Hydrogen bonding and electrostatic interactions between the perilla protein and alginate molecules were confirmed using Fourier-transform infrared (FTIR) spectroscopy. The authors concluded that the samples with this formulation had better antimicrobial properties, release profiles, and thermal stabilities than the control samples, and can be used to increase the shelf life of food products (Qiu et al. 2023).

Nanoliposomes have also been used to encapsulate REOs for food and cosmetic applications. For instance, Wen et al. (2010) incorporated REOs into liposomes by rapid expansion of a supercritical solution of lipid materials and REOs. They reported that the resulting REO-loaded liposomes were relatively small (< 100 nm). They also reported that using this method gave good controlled release, smaller particle size, and higher encapsulation efficiency (Wen et al., 2011).

## 5 Biological activities of REOs

REOs have a broad range of biological activities, including antimicrobial, antioxidant, therapeutic, pigmentation, and aromatic properties. In this section, we focus on the first three of these bioactivities.

### 5.1 Antimicrobial properties

REOs contain a variety of components that exhibit antimicrobial properties. For instance, kaempferol and quercetin glycosides have been reported to inhibit bacteria, whereas *E. coli* and *S. aureus* (Androutsopoulou et al., 2021), whereas heneicosane alkanes, nonadecane, geraniol, and α-pinene have been reported to inhibit fungi (Ghavam et al., 2021). Halawani (2014) studied the antimicrobial effects of alcoholic and aqueous extracts of roses against *S. aureus, P. aeruginosa, E. coli, Streptococcus pneumoniae, Acinetobacter calcoaceticus, Salmonella enteritidis,* and *Aspergillus niger* and found that both extracts inhibited their growth. The ethanol extract showed the most potent effect against *P. aeruginosa*, with a minimum inhibitory concentration (MIC) and minimum bactericidal concentration (MBC) of 62.5 g/mL (Halawani, 2014). In another study, Trendafilova et al. (2023) characterized the antimicrobial effects of aqueous ethanol and ethyl acetate extract from rose flowers against *Propionibacterium acnes*, *S. aureus, S. epidermidis*, and *Candida albicans*. Their research showed that the extracts inhibited bacterial growth (by 100 %) but not fungal growth (Trendafilova et al., 2023). Similarly, Ghavam et al. (2021) studied the antimicrobial properties of REOs by measuring the inhibition zones, and found that they exhibited good antibacterial effects against *S. aureus* (IZ; 11.33 mm), *K. pneumonia* (IZ; ∼ 8.00 mm with MIC of 500 μg/mL), and *Streptococcus pyogenes* (IZ: 9.33 mm), and good antifungal effects against *Aspergillus brasiliensis* (IZ; 15 mm) and *C. albicans* (IZ; 6 mm with MIC of 125 μg/mL). In this case, citronellol, geraniol, and α-pinene were reported to play an important role in their antimicrobial activity. The antifungal properties of REO can be attributed to oxygenated compounds, such as linalool (Ghavam et al., 2021). Moreover, Chroho et al. (2020) reported that a aqueous ethanol extract of rose had antibacterial effects against *E. coli, Salmonella typhimurium*, *S. aureus*, and L. *monocytogenes* (Chroho et al., 2022). In addition, Niazi et al. (2023) illustrated the potent antimicrobial action of ethanolic rose extracts against *Bacillus subtilis, S. aureus, E. coli, Aspergillus fumigatus, Aspergillus niger, C. albicans*, and *Monascus purpureus* (Niazi et al., 2025).

The antimicrobial properties of REOs arise not only from their major and primary ingredients but also from the synergistic interactions of minor constituents present in smaller levels. These synergistic effects contribute to different biological activities, primarily by compromising the permeability and integrity of bacterial cell membranes. This disruption can lead to critical changes in the cytoplasmic contents, ultimately causing membrane-dependent conduction systems to malfunction or rupture (Ghavam et al., 2021).

### 5.2 Antiviral activity

The antiviral activity of *rosa damascena* is typically driven by its diverse bioactive compounds, including flavonoids (e.g., quercetin, kaempferol), phenolic acids, and essential oils (e.g., geraniol, citronellol). These compounds exert antiviral effects through specific molecular mechanisms that target various stages of the viral life cycle and host responses (Vilhelmova-Ilieva et al., 2021).

For instance, Mahmood et al. reported that both water and methanol extracts of roses possessed substantial anti-HIV activities. Researchers have identified several active compounds in rose that responsible for anti-viral effect: (1) kaempferol, which selectively inhibits the viral protease, (2) quercetin, which prevents the binding of gp120 to CD4, and (3) 2-phenylethanol-O-(6-O-galloyl)-β-D-glucopyranoside, which irreversibly interacts with gp120. These findings highlight the potential of roses extracts as a source of natural antiviral agents (Mahmood et al., 1996). Moreover, Vilhelmova-Ilieva et al. (2021) observed that REOs inhibited the replication of Victoria strain and R-100 strains by 20 and 10 %, respectively (Vilhelmova-Ilieva et al., 2021). In another study, Androutsopoulou et al. (2022) investigated the antiviral properties of REOs as natural preservatives against adenovirus 35. They found that a 5 % concentration of REOs significantly prevented the growth of this virus (Androutsopoulou et al., 2021). Research suggests that flavonols within REOs play a crucial role in blocking viral entry into host cells by interacting with viral attachment factors and/or membrane fusion proteins. Moreover, these compounds suppress the signaling pathways essential for viral gene expression, inhibit remodeling enzymes and channels that regulate viral movement, and reduce the transcription of the viral genome along with viral protein synthesis (Zakaryan et al., 2017).

The following sections detail these mechanisms to address current knowledge gaps and provide a foundation for future research. Flavonoids (e.g., quercetin, kaempferol) and essential oils (e.g., geraniol, citronellol) from *rosa damascena* exhibit antiviral effects through multiple mechanisms: (i) inhibiting viral entry by blocking spike protein interactions with host receptors like ACE2 (Meng et al., 2023). (ii) disrupting viral replication by targeting enzymes like RNA-dependent RNA polymerase and host signaling pathways, thereby limiting viral proliferation (Mustafa et al., 2023; Prosvetova et al., 2023). (iii) enhancing immune responses by upregulating interferon-stimulated genes and reducing oxidative stress (Baranwal et al., 2021; Fast et al., 2019). (iv) impairing viral assembly and release by altering envelope fluidity and inhibiting host proteases (Mustafa et al., 2023).

**Fig. 4:**
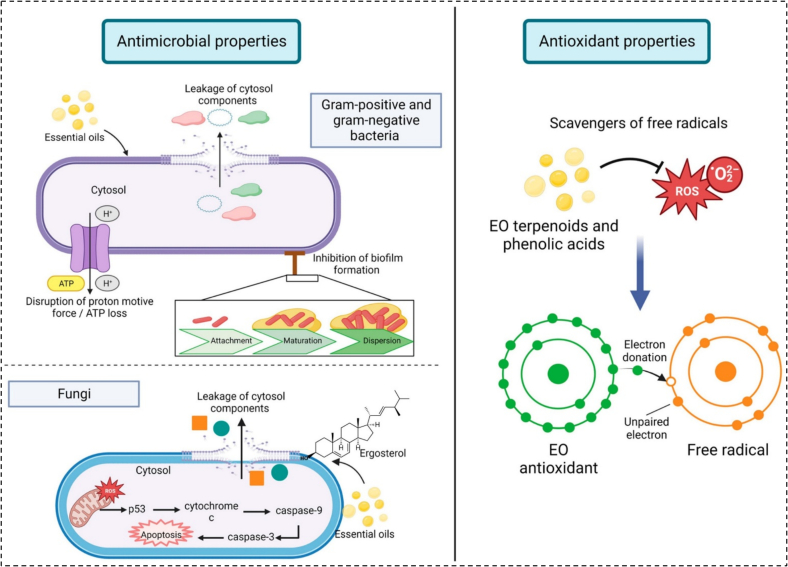
Schematic mechanism of antimicrobial and antioxidant effects of REO (Chávez-Delgado & Jacobo-Velázquez, 2023). Copyright © 2023, MDPI.

Future research should use molecular docking, proteomic/transcriptomic analyses, and in vitro/in vivo studies to validate these mechanisms and explore synergistic effects for antiviral applications in functional foods and therapeutics. Details about the antimicrobial effects of REO are summarized in schematic form in Fig. 4.

### 5.3 Antioxidant properties

Roses are rich sources of polyphenols and flavonoids that exhibit strong antioxidant activity (Liu et al. 2020). For instance, Liu et al. (2020) reported that rose flower residues at 2.760 and 2.258 μg/mL (IC_50_) were able to scavenge DPPH and ABTS radicals, due to their high content of phenolic compounds such as quercetin, kaempferol, and gallic acid, which reduce the formation of free radicals and reactive oxygen species (ROS) (Liu et al. 2020). Additionally, Chroho et al. (2020) reported that an aqueous ethanol extract of rose flowers, with an EC₅₀ of 0.20 mg/mL, exhibited significant antioxidant activity, scavenging free radicals with a capacity of 213.22 mg AAE/g and a ferric reducing power of 164.23 μM Fe(II) (Chroho et al., 2022). Moreover, Zahedi-Amiri reported that rose extracts decreased aluminum chloride-induced oxidative stress in rats' blood serum and protected the cells against reactive free radicals (Zahedi-Amiri et al., 2019). In a study by Mawarni et al. (2020), the antioxidant activities of rose petal extract (RPE) and rose receptacle extract (RRE) were evaluated, and reported that RPE exhibited greater DPPH free radical scavenging activity (IC₅₀ = 4.46 μg/mL) than RRE (IC₅₀ = 15.49 μg/mL). This stronger activity was attributed to the higher concentrations of proanthocyanidins and anthocyanins in the petal extract (Mawarni et al., 2020). Önder (2023) examined the antioxidant and enzyme activity in the petals of oil-bearing roses at five distinct stages of flower development. The results indicated that the antioxidant enzyme activity and free radical scavenging potential were significantly greater in the bud stage (Stage I) than in the fully opened flowers (Stage V). This variation can be attributed to differences in developmental stages, where an increase in reactive oxygen species (ROS) accumulation is linked to a decrease in antioxidant activity (Önder, 2023).

### 5.4 Therapeutic properties

Rose extracts have been reported to possess anti-inflammatory, antidiabetic, anticancer, cardioprotective, hepatoprotective, cardioprotective, and neuroprotective properties. For instance, Yari et al. (2024) investigated the effects of REOs on breast inflammation. They observed that REOs stimulated apoptosis in a model human breast cancer cell line (MDA-MN-231), suggesting that they may be beneficial for the treatment of patients with breast cancer (Yari et al., 2024). Shokrzadeh et al. (2017) reported that a dose of 10 μg/mL of REOs exhibited anticancer effects (Shokrzadeh et al., 2017). REOs and their extracts have also been reported to exhibit health benefits used in aromatherapy applications (Dagli et al., 2019). Davoodi et al. (2017) reported that daily supplementation with an aqueous alcoholic extract of rose significantly reduced high-density lipoprotein (HDL) levels, liver fat accumulation, and hepatic enzyme levels in a high-fat diet-induced nonalcoholic fatty liver disease (NAFLD) rat model after six weeks of treatment. These results suggest that dietary supplementation with rose extracts holds potential therapeutic promise for the treatment of NAFLD. The primary pharmacological mechanism driving this effect was attributed to an amelioration of oxidative stress-related injury in the liver tissue (Davoodi et al., 2017).

Rose extracts have also been reported to inhibit alpha-amylase and alpha-glucosidase activity, which may retard starch digestion, thereby having beneficial effects on human nutrition by inhibiting blood glucose spikes. Indeed, Alsalti et al. (2023) reported that aqueous and methanolic extracts of rose petals could exhibit antidiabetic properties in diabetic mice by reducing carbohydrate digestion and absorption in the intestine and so lowering postprandial blood glucose levels (Alsalti et al., 2022). Rose petal extracts may also have beneficial effects on cardiovascular health. For instance, Fathima and Murthy (2019) highlighted the cardioprotective effects of rose extracts using a rat model of myocardial infarction induced by isoproterenol (Fathima & Murthy, 2019).

## 6 Applications of REOs

REOs have found a broad spectrum of applications in the food and pharmaceutical industries, and some of the most important ones are highlighted in this section.

### 6.1 Foods and beverages

#### 6.1.1 Food preservation: antimicrobial and antioxidant activities

REOs and their extracts have been used as natural preservatives in meat, seafood, fruit, vegetable, confectionery, and other products because of their antibacterial, antifungal, insecticidal, and antioxidant properties. For example, Saffari et al. (2023) studied the effects of REOs-loaded nanoemulsion (at 75. 125, and 250 μg/g) on preserving ground sheep meat when stored at 4 °C for 12 days. They found inhibition of *lactic acid bacteria*, *S. typhimurium*, and *coliforms*, a reduction in pH and total volatile basic nitrogen (TVB-N) levels, as well as an increase in sensory scores of flavor and overall acceptability for up to 12 days, thereby indicating the potential of the rose extracts as preservatives for meat-based products. Giannakourou et al. (2019) examined the combinatory impact of an osmotic treatment and rose extracts on the shelf life and quality of sea bass fillets during refrigerated storage at 5 °C. They found that this combined treatment inhibited the growth of spoilage bacteria and increased the shelf life of the product by 4-fold.

In fruits and vegetables, Verešová et al. (2024) reported that vapor from REOs could suppress microbial growth in kiwi and banana models. This volatile essential oil also exhibited a strong antibacterial effect on the microbiota of *sous vide* processed eggplant, inhibiting the growth *Salmonella enterica* during storage. Also, it showed potent insecticidal activity, producing a 70 % mortality against *Megabruchidius dorsalis.*

In bakery products, Chochkov et al. (2022) studied the effects of cocoa pod husks (CPH) and *R. damascena* by-product (RDCO_2_), obtained after supercritical CO_2_ extraction, as biopreservatives and functionalizing additives in muffins. They observed that these natural phytochemicals increased dietary fiber content by 3 times, polyphenol content by 2.5 times, and extended the muffin shelf life to 20 days at 22 °C. The batter containing CPH and RDCO₂ exhibited the highest yield stress (7.18 Pa) and the finest texture, confirmed by 75.6 % of gas pores being smaller than 1 mm^2^. Sensory analysis showed that muffins with these additives maintained high acceptability, characterized by a pleasant taste and distinctive fragrance.

Rose petals contain significant levels of nitrate that could be explored as a partial replacement for sodium nitrite (E250) in cured meat product processing (Kolev, 2022; Konteles et al., 2023). The rose to decreased the growth rates of aerobic and lactic acid bacteria. In addition, replacing half of the nitrites with rose extract in bacon maintained its red color and sensory quality and effectively increased its shelf life (78 days) compared to the control sample (60 days). The researchers therefore concluded that rose extract could be used as a natural antimicrobial in the production of cured meat products with low nitrite usage (Konteles et al., 2023).

In confectionery products, Akhavan and Mehrizi (2016) studied the preservative effects of rose extract (0.1, 0.3, and 0.5 %) in Sohan (an Iranian confectionary product). These samples were packaged and stored at 25 °C for 180 days. The sample containing 0.5 % rose extract showed meaningly better antioxidant (24.6 %), antifungal (<10 CFU/g), and sensory properties. The 0.5 % rose extract had a significant inhibitory effect on rancidity for 90 days storage.

#### 6.1.2 Food flavor and colorant agent

Flavoring and coloring agents are often added to foods to improve their sensory appeal (Wu et al., 2022). Nevertheless, growing concerns about the potential adverse health effects of artificial additives in foods have prompted interest in the development of natural alternatives (Getachew et al., 2019; Zang et al., 2023). Several researchers have shown that rose extracts can be used as natural functional ingredients in food products.

For example, Pires et al. (2018) investigated the use of various flower extracts (rose, cornflower, and dahlia) as replacements for the artificial yellow orange colorant (E163) in yogurt. The study observed that yogurt samples formulated with rose extract exhibited no significant difference in red color intensity (a value) compared to those with E163. Nutritionally, all yogurt samples had highly similar profiles, with water constituting the largest component, followed by carbohydrates and proteins. Moreover, the rose-extract yogurt showed a stable fatty acid composition, with particularly with higher percentages of C14:0 and C16:0, after 7 days of storage. Therefore, the rose extract could be successfully used to replace the E163 in this product. In another study, Hadian et al. (2023) encapsulated REO into β-cyclodextrin using a precipitation method, which was then used in a beverage formulation. A formulation containing only the REO/β-CD complex showed an initial antioxidant activity with an IC_50_ of 1663.7 μg/100 mL, which increased to 2011.4 μg/100 mL after 40 days of storage at room temperature. Microbial counts remained below 100 CFU/mL, under the Iranian standard limit of 200 CFU/mL. Sensory evaluation showed this formulation had a significantly better taste (8.2) compared to other formulations, with no significant differences in color, appearance, or aroma. The study by, Kumar et al. (2021) used rose syrup (10 %) and marigold powder (0.5 %) as a functional additive, for a flavoring and coloring, in a milk beverage formulated with nutricereals (finger millet and oats). The results showed that these rose-flavored milk beverages was a good source of bioactive compounds, indicating high total phenols (119.18–145.23 %), β-carotene (0.37 %), anthocyanins (78.82–230.58 %), and DPPH free radical scavenging (4.98–7.17 %). Sensorily, the milk beverage was highly acceptable (a score of 7.83) and remained stable for 50 days when stored at 4 °C.

### 6.2 Food packaging materials

Active packaging materials are typically polymeric films containing additives that can enhance the shelf life and quality of packaged foods (Sani et al. 2024). REO and extracts have been explored as potential film additives for these applications because of their antioxidant and antimicrobial activities. For example, Liu et al. (2023) developed nanocomposite films with good antioxidant and antibacterial properties using REO and chitosan/chitin nanofibers as additives. These films were shown to effectively extend the storage life of food products. Aghajani-Memar et al. (2024) developed REO-loaded sodium caseinate/halloysite nanotube/bacterial cellulose films and showed they could be used as antibacterial biodegradable packaging materials to preserve perishable food products. Xue et al. (2024) developed REO-loaded soy protein/polyphenol emulsion-based films and showed that they extended the shelf life of cherry tomatoes.

Other researchers have also shown that rose extracts can also be used in active packaging coatings designed to enhance food preservation. Monfared et al. (2024) evaluated the effects of zedo gum coatings loaded with emulsified damask rose extract on the storage quality of Beluga sturgeon fillets, and found that they could be used to successfully inhibit microbial growth and improve sensory attributes throughout storage. The layer-by-layer deposition technology has been used to encapsulate essential oils in intermediate layers to reduce their loss. Improvements in the retention and sustained release of these encapsulated essential oils demonstrates the potential of these coatings for food packaging applications (Zhang et al., 2022). In another study, Akbari et al. (2022) prepared a layer-by-layer coating with rose extract and probiotics as antibacterial agents, carrageenan-rose extract as the inner layer, and chitosan and *Bacillus coagulans* as the outer layer for walnuts. The population of bacteria was above 8.041 Log Cfu/g after 49 days of storage, confirming the probiotic potential of walnuts coated with these kinds of multilayer films.

Anthocyanins in roses exhibit changes in color when the pH of their surroundings changes, appearing red at acidic pH, violet or purple at neutral pH, and blue at alkaline pH (Wahyuningsih et al., 2017). Consequently, they can be used as natural pH indicators in smart packaging materials (Maqsood-ul-Haque & Shariffuddin, 2022). For instance, changes in the pH of foods can occur due to fermentation or deterioration, which can be detected using these natural colorimetric sensors. Rose anthocyanins have successfully been used as pH indicators in polymer films and coatings for tracking the freshness of perishable foods, such as fish, shrimp, poultry, and meat (Table 3). For instance, Jiao et al. (2024) prepared pH-sensitive films from carrageenan gum and maize starch that contained a colored rose extract as a visual indicator of pork freshness. These films were shown to exhibit significant color changes when exposed to different pH values and or ammonia solutions. Similarly, Wu et al. (2023) developed a corn-starch and chitosan-based aerogel containing rose anthocyanins encapsulated in potato amylopectin nanoparticles and hydrophilic silica. Changes in the color of these aerogels during storage at 4 °C for six days were used to provide information about shrimp quality changes. Likewise, Thakur et al. (2023) developed pH-sensitive films by incorporating rose petal extract and critic acid into a buckwheat starch matrix. These anthocyanin-containing films revealed a color change from pinkish to greenish-yellow when the pH increased from 5 to 9. In another study, Qiu et al. (2023) produced edible pH-sensitive films based on rose anthocyanin integrated into sodium alginate and apricot peel pectin films and then showed they could be used to monitor the freshness of grass carp during storage at 4 °C for 14 days. Although the addition of rose anthocyanins weakened the mechanical strength and water vapor permeability of the films, they provided colorimetric films with excellent free-radical scavenging and UV-blocking abilities. The potential practical application of these films was then assessed by using them as packaging materials for grass carp meat. A color shift from light pink to light yellow and dark yellow occurred during storage indicating deterioration in the quality of the fish. In addition, Wang et al. (2022) fabricated an active/smart film based on carboxymethyl cellulose-polyvinyl alcohol with rose petal extract that could monitor the freshness of Indian mackerel. The addition of the rose extract reduced the water solubility, moisture, swelling, water vapor transmission rate, and elongation at break of the films, and provided good antioxidant and antibacterial activities. During food spoilage, the indicator film showed red-to-green color shifts after 12 days of storage, indicating quality deterioration.

**Table 3:** Functional properties and biological activities of rose anthocyanin-based active/intelligent packaging films.

**Film properties**		**Functional properties**		**Film characterization**			**Ref.**
***Polymer***	***Content***	**In vitro *observations***	***Practical applications (Food model/color response)***	***Physical and Mechanical properties***	***Barrier Properties***	**Functional properties**	
CA/CMC/RA	0, 2.5, 5, 7.5, 10 %	pH value = 2–11/red to yellow-green	Hairtail/color changed from pink to yellow	TS ↓ (11.7–10.6 MPa) EBA ↑ (15.34–17.78 %)	WVP ↑	AA ↑ DPPH (6.30–84.93 %; IC_50_, 0.064 mg/ml) ABTS (10.71–95.29 %; IC_50_, 0.097 mg/ml)	(Wang et al., 2024)
SA/CMC-Na/RA	2, 4, 6, 10, 20, 30 %	pH value = 2–12/ bright red to yellow	*Penaeus vannamei*/color changed from pink to yellowish green	MC ↑ (15.54–20.69 %) WCA ↑ (39.65–46.91°) TS ↑ (15.61–16.50 MPa) EBA ↓ (8.20–5.38 %)	WVP ↑ (8.89–11.07 × 10^−10^ g·(cm·s·KPa)^−1^)	NA	(Yang et al., 2022)
BS/citric acid/RA	5–18 %		NA	MC ↓ (14.05–11.49 %) TS ↑ (1.75–8.03 MPa) EBA ↓ (5.69–1.30 %) WS ↓ (45.12–31.4 %)	WVTR ↑↓ (2.01–2.01 × 10^−06^, g/m^2^/24 h)	AA (DPPH) (up to 96.85 %)	(Thakur et al., 2023)
CMC-Na/PVA/RA	0, 40, 80 and 160 mg/100 mL	pH value = 1–14/rose red to dark yellow	Pork/color change from light-green to dark green-orange	MC ↓ (10.87–10.57 %) SI ↓ (286.21–278.59 %) WS ↓ (27.85–26.94 %)	WVP ↑ (8.39–9.42 × 10^−11^ g. m/m^2^. s. Pa)	AA ↑ DPPH (65.74–83.80 %) ABTS (20.57–57.86 %)	(Wang et al., 2022)
CA/RP-CDs/RPA	5.0 %	pH value = 2–12/ red to blue	Minced pork and shrimp/color change from red to dark yellowish	WCA ↑ (41.1–49.2°) TS ↑ (46.9–59.7 MPa) EBA ↑ (3.8–5.1 %)	WVP ↓ (6.14–3.27 × 10^−9^ g.m/m^2^.Pa.s)	AA ↑ DPPH (5.28–78.25 %) ABTS (7.26–86.54 %)	(Wagh et al., 2024)
PVA/OMP/RA	20, 25 and 30 mL/100 mL	pH value = 2–12/ red to yellow-green	Shrimp/ color change from purple to yellow	TS ↑ (26.5–36.0 MPa) YM ↑ (3.2–11.1 GPa) EBA ↓ (176 → 110.9 %)	WVP ↓ (7.7–5.9 × 10^−7^ g.mm^−2^ s^−1^ atm^−1^) OP ↓ (0.92–0.63 × 10^−5^ cm^3^mm^−2^ d^−1^ atm^−1^)	NA	(Kang et al., 2020)
CH/GA/RA	5 %	pH value = 1–12/ pale pink to brown	Chicken fillets/ color change from white to khaki	WS (32.17–19.56 %) MC ↓ (5.42–2.21 %) TS ↓ (6.71–3.77 MPa) EAB ↑ (7.68–111.43 %)	WVP ↓ (18.97–9.03 × 10^−4^ g mm/m^2^ h Pa)	AA (DPPH) ↑ (up to 36.19 %)	(Shavisi & Shahbazi, 2022)
Starch/CH/ APNPs	0, 40,60, 80, 100	pH value = 2–12/ light red to dark yellow	Shrimp/color change from pink to yellow	TS ↑ (11.17–25.85 MPa) EAB ↓ (28.80–4.87 %)	WVP ↓ (12.24–7.01 × 10^−12^ g m^−1^ s^−1^ Pa^−1^)	NA	(Zheng et al., 2023)
CMC/PVA/RPA	5 %	pH value = 2–10/ bright red to yellow	Indian Mackerel	MC ↓ (11.92–11.05 %) TS ↑ (12.01–14.78 MPa) EB ↓ (37.52–35.21 %)	WVTR ↓ (2360–1654 g.m^−2^ day^−1^)	AA (DPPH) ↑ (up to 78.86 %) Antibacterial effect on *P. fluorescens*	(Kanatt, 2021)

CA: Carrageenan; CMC: Carboxymethylcellulose; RA: Rose anthocyanin; SA: Sodium alginate; PVA: Polyvinyl alcohol; CDs: Carbon dots; RPA: Rosa petal anthocyanin; OMP: Okra mucilage polysaccharide; CH: Chitosan; GA: Gum Arabic; BS: Buckwheat starch; APNPs: Potato amylopectin nanoparticles; MC: Mositure content; WS: Water solubility; WCA: Water contact angle; EBA: Elongation at break; TS: Tensile strength; WVTR: water vapor transmission rate; WVP: Water vapor permeability; AA: antioxidant activity; ↑: Increase; ↓: Decrease, ↑↓: Variable; NA: not analyzed.

### 6.3 Pharmaceutical and medical applications

Different parts of roses, can be used for both cosmetic and therapeutic purposes, such as fruits, flowers, leaves, roots, and bark (Gunawardana & Jayasuriya, 2019). Roses contain a variety of phenolic compounds, which have been linked to therapeutic effects (Boskabady et al., 2011). The following section provides examples of evidence-based applications of rose extracts for the treatment of various medical conditions.

#### 6.3.1 Sleep disorders

Multiple studies have evaluated the role of aromatherapy using different rose formulations for improving sleep quality (Ghorbani Rami et al., 2021). In a randomized clinical trial (RCT), Mokhtari et al. (2023) 60 burn patients were divided into intervention and control groups. The intervention group received three nights of five drops of 40 % REO, while the control group received distilled water. Using the St. Mary's Hospital Sleep Quality Questionnaire, the REO group showed significantly better sleep quality and lower anxiety. Another RCT with 80 operating room personnel during COVID-19 compared REO inhalation to a paraffin oil placebo, finding improved sleep quality in the REO group via the Pittsburgh Sleep Quality Index (PSQI) (Mahdood et al., 2022).

#### 6.3.2 Anxiety and depression

The role of REO in reducing anxiety and alleviating depressive symptoms has also been evaluated in numerous studies. In the previously mentioned RCT on operating room personnel during COVID-19, REOS inhalation significantly lowered anxiety scores (Mahdood et al., 2022). Another single-blind RCT involving patients undergoing coronary angiography showed that inhaling five drops of 40 % REO for 20 min significantly reduced stress, anxiety, and hemodynamic parameters compared to distilled water placebo, suggesting its suitability for such patients (Bikmoradi et al., 2022). In contrast, in a placebo-controlled RCT, Hosseini et al. (2024) found no significant effect of REO aromatherapy on postpartum depression. In a meta-analysis of RCTs, Rasooli et al. (2021) found that administration of REOs could significantly reduce adult anxiety, depression, and stress, but the quality of the included studies was only considered “fair” based on the Cochrane risk of bias assessment tool (Rasooli et al., 2021).

#### 6.3.3 Gastrointestinal and periodontal disorders

REOs and other extracts have also been used to treat various gastrointestinal and periodontal disorders. A double-blind RCT on 100 children over 12 months compared REO syrup (with brown sugar) to polyethylene glycol (PEG) (Imanieh et al., 2022). After four weeks, the REO group achieved a 100 % cure rate versus 91.7 % for PEG, with an odds ratio of 1.09, though not statistically significant, suggesting REO as a safe, cost-effective alternative. Another RCT with 160 adults (age range from 18 to 75 years) found REOs as effective as lactulose for chronic constipation (Sadeghi Mansoorkhani et al., 2021). A double-blind RCT on 96 patients with constipation-predominant irritable bowel syndrome (IBS-C) tested a Persian herbal formula (MPR) containing REOs, *Melissa officinalis*, and *Pimpinella anisum* (Azimi et al., 2024).

#### 6.3.4 Migraine and other pain related disorders

REO has also been investigated for treatment of migraine and other pain related disorders. For instance, a double-blind, placebo-controlled crossover trial investigated the efficacy of topical REOs in 40 patients with migraine (Niazi et al., 2017). The study employed a cross-over design with a washout period. REO significantly reduced pain intensity in patients with “hot” type migraine compared to “cold” type, but showed no difference in nausea, vomiting, photophobia, or phonophobia severity, suggesting short-term pain relief for specific migraine subtypes.

Other researchers have carried out an RCT to investigate the effectiveness of REO aromatherapy at reducing pain and anxiety during the first stage of labor in nulliparous women (Hamdamian et al., 2018). The participants received either REO aromatherapy or saline solution every 30 min. The results showed significantly lower pain scores and anxiety levels in the aromatherapy group than in the control group. Although no significant differences were observed in labor duration or delivery method, these findings indicate that REO aromatherapy is an effective, non-pharmacological intervention for mitigating pain and anxiety in the early stages of labor.

#### 6.3.5 Endocrine effect and sexual function

A RCT evaluated REOs for sexual dysfunction in 50 men (mean age 40) with opioid use disorder (Farnia et al., 2017). Participants were assigned to receive REO drops or placebo, with sexual and erectile function assessed at baseline, four, and eight weeks, alongside testosterone levels. The REO group showed reduced sexual dysfunction and increased testosterone compared to placebo, though improvements were not consistently linked to testosterone levels, suggesting REO's potential to improve sexual function in this population.

Selective serotonin reuptake inhibitors (SSRIs) commonly cause sexual dysfunction, which is a significant drawback in treating major depressive disorder (MDD). A double-blind, placebo-controlled RCT evaluated the effects of rose essential oil (REO) on SSRI-induced sexual dysfunction in 60 men (mean age 32) with major depressive disorder (MDD). Participants were divided into groups receiving either REO or a placebo. Self-assessed measures of depression and sexual function were collected at baseline, four, and eight weeks (Farnia et al., 2015). The REO group exhibited a significantly greater reduction in sexual dysfunction, with notable improvement occurring between weeks four and eight, as well as a more substantial improvement in depressive symptoms compared to the placebo group. These results suggest REO's potential as an adjunctive treatment for alleviating both SSRI-related sexual dysfunction and depressive symptoms in men with MDD.

## 7 Safety aspects of REO

The widespread use of *R. damascena* in food and health products necessitates a thorough evaluation of its safety and toxicity across all its forms and applications. Acute and subacute toxicity studies, including one by Batool et al., found *R. damascena* extracts to have low toxicity in mice. The oral doses of up to 1000 mg/kg for 14 days caused no adverse effects on blood parameters or organ histology, supporting a high in vivo safety (Batool et al., 2018). Similarly, a trial in a rabbit model evaluated the oral administration of arq-e-Gulab (AG) infusion at two doses (250 and 500 mg/kg) over 60 consecutive days. The findings showed no nephrotoxic effects, with serum creatinine levels remaining unaltered, a safety profile potentially linked to its antioxidant constituents like quercetin and ellagic acid (Osama et al. 2022). Hydrosols of R. damascena showed low cytotoxic and genotoxic effects at high concentrations (3–20 %, 4 h). They also reported a potent cytoprotective and genoprotective effect against the mutagen MNNG, expressed by a more than twofold reduction in chromosomal aberrations and micronuclei (Gateva et al., 2023).

Despite this strong evidence of the plant's intrinsic safety, quality control of commercial products is paramount. A study of rose water brands from Tehran markets aimed to identify methanol and ethanol content, noting that while these toxic compounds can cause significant complications if present in high concentrations, the findings revealed that the mean levels were significantly lower than the Maximum Residue Levels, posing no health risk (Yazdanfar et al., 2022). As a valuable plant-derived aromatic product with versatile uses in human life, *R. damascena* Mill. essential oil should be used in an appropriate concentration range tailored to cellular sensitivity.

## 8 Conclusion and perspective

This review has explored into the phytochemicals of *Rosa damascena* essential oils (REOs), featuring over 200 bioactive compounds like flavonoids (such as quercetin and kaempferol), essential oils (including geraniol and citronellol), and phenolic acids. These elements contribute to potent antioxidants, antimicrobial, and anti-inflammatory activities, with mechanisms that inhibit microbial growth by disrupting cell membranes, scavenge free radicals to prevent oxidation, and modulate inflammatory pathways. In the food sector, REOs stand out as natural preservatives, extending shelf life in products like oils, meats, and beverages by inhibiting spoilage bacteria and lipid peroxidation; they also serve as functional ingredients in nutraceuticals, enhancing nutritional profiles with antioxidant improvements and flavor enhancement in clean-label foods. While healthcare applications investigate anti-inflammatory and antiviral potentials, the core emphasis here is on food safety and preservation, where REOs offer safer alternatives to synthetic additives among rising consumer demand for natural options. Nonetheless, challenges remain, such as optimizing extraction for food-grade purity, ensuring stability in formulations, exploring synergies for enhanced preservation efficacy, and conducting stability tests under real food processing conditions.

Moving forward, to determine *rosa damascena's* role in the food industry, future efforts should focus on targeted advancements. First, sensory and stability studies in food matrices; incorporating REOs into dairy, bakery, or packaged foods will validate their preservative effects, flavor integration, and shelf-life extension without off-tastes, using standardized concentrations for regulatory approval. Second, refining green extraction techniques, like ultrasound-assisted or microwave methods, will increase yields of key bioactives while ensuring sustainability, scalability for industrial food production, and minimal environmental impact. Third, applying omics approaches (metabolomics and proteomics) alongside molecular modeling will clarify how these compounds interact with food pathogens and oxidants, uncovering synergies such as blending flavonoids with essential oils for superior antimicrobial activity in functional foods. Finally, in vitro simulations of food environments and pilot-scale studies on encapsulation for better delivery in products will bridge gaps, complemented by sustainable cultivation research to secure supply chains. Coupled with consumer education on natural preservatives and ethical sourcing, this will position REOs as a candidate for innovative, health-oriented food systems, aligning with global trends toward clean, sustainable eating and reducing reliance on artificial additives.

Moreover, to advance research on phytochemicals and bioactive functional ingredients from *Rosa damascena*, future studies should focus on addressing specific gaps in the current literature. (i) clinical research should prioritize well-designed, randomized controlled trials to evaluate the efficacy and safety of *Rosa damascena*-derived compounds in targeted health conditions, such as inflammation, oxidative stress, and metabolic disorders, with standardized dosages and formulations. (ii) optimization of extraction techniques should explore novel, sustainable methods, such as ultrasound-assisted or enzyme-assisted extraction, to enhance the yield and stability of bioactive compounds like polyphenols and essential oils, while minimizing environmental impact. (iii) research should investigate the scalability of these extraction methods for industrial applications in the food and healthcare sectors, with a focus on cost-effectiveness and regulatory compliance. (iv) studies should aim to elucidate the molecular mechanisms underlying the bioactivity of *Rosa damascena* compounds through advanced technologies to identify potential therapeutic targets and improve their application in functional foods and nutraceuticals. Therefore, sustainable practices in cultivation and increased consumer education are essential for promoting its benefits and ensuring responsible use. Overall, REO demonstrates a promising future in supporting health and well-being, while fostering sustainable practices in its industry.

## CRediT authorship contribution statement

**Arezou Khezerlou:** Writing – original draft, Methodology, Investigation, Conceptualization. **Keyhan Mohammadi:** Writing – original draft, Investigation. **Amirhossein Abedini:** Writing – original draft, Validation, Investigation. **Maryam Alizadeh Sani:** Writing – review & editing, Methodology, Investigation, Conceptualization. **Mahmood Alizadeh Sani:** Writing – review & editing, Methodology, Investigation, Conceptualization. **David Julian McClements:** Writing – review & editing, Methodology, Investigation.

## Funding

No funding was received.

## Declaration of competing interest

The authors declare that they have no known competing financial interests or personal relationships that could have appeared to influence the work reported in this paper.

## Data Availability

The data that has been used is confidential.

## References

[bb0005] Aghajani-Memar S., Hamedi S., Kermanian H. (2024). Preparation of the Edible Fragrant Antibacterial Sodium Caseinate–Based Nanocomposite Containing Rosa damascena Essential Oil and Halloysite Nanotube. Food and Bioprocess Technology.

[bb0010] Ahadi H., Shokrpour M., Fatahi R., Naghavi M.R., Mirjalili M.H. (2023). Essential oil, flavonoids and anthocyanins profiling of some Iranian damask rose (Rosa damascena mill.) genotypes. Industrial Crops and Products.

[bb0015] Akbari E., Parastouei K., Abbaszadeh S. (2022). Physico-chemical and sensory analysis of walnut coated with rose extract and probiotic: a layer-by-layer approach. Journal of Food Measurement and Characterization.

[bb0020] Akhavan H., Mehrizi R.Z. (2016). Effects of damask rose (Rosa damascena mill.) extract on chemical, microbial, and sensory properties of Sohan (an Iranian confection) during storage. Journal of Food Quality & Hazards Control.

[bb0025] Akram M., Riaz M., Munir N., Akhter N., Zafar S., Jabeen F., Altaf S.H. (2020). Chemical constituents, experimental and clinical pharmacology of Rosa damascena: a literature review. Journal of Pharmacy and Pharmacology.

[bb0030] Alizadeh Z., Fattahi M. (2021). Essential oil, total phenolic, flavonoids, anthocyanins, carotenoids and antioxidant activity of cultivated damask rose (Rosa damascena) from Iran: With chemotyping approach concerning morphology and composition. Scientia Horticulturae.

[bb0035] Alsalti A.A., Hasan N., Aldia D. (2022). In-vitro and in-vivo hypoglycemic efficacy of Rosa damascena petals extracts. Bulletin of Pharmaceutical Sciences Assiut University.

[bb0040] Androutsopoulou C., Christopoulou S.D., Hahalis P., Kotsalou C., Lamari F.N., Vantarakis A. (2021). Evaluation of essential oils and extracts of rose geranium and rose petals as natural preservatives in terms of toxicity, antimicrobial, and antiviral activity. Pathogens.

[bb0045] Anmol G.A., Shivani M.K., Gupta M.S., Sharma U. (2025). Natural deep eutectic solvents-based concurrent approach for qualitative and quantitative enhancement of Rosa damascena essential oil and recovery of phenolics from distilled rose petals. Separation and Purification Technology.

[bb0050] Azimi M., Shahrbabaki H.K.D., Raeiszadeh M., Eslami O. (2024). Effects of a traditional herbal formula containing Melissa officinalis, Pimpinella anisum, and Rosa damascena on anxiety and depression in patients with constipation-predominant irritable bowel syndrome (IBS-C): A double-blind randomized clinical trial. Explore (New York, N.Y.).

[bb0055] Baranwal M., Gupta Y., Dey P., Majaw S. (2021). Antiinflammatory phytochemicals against virus-induced hyperinflammatory responses: Scope, rationale, application, and limitations. Phytotherapy Research.

[bb0060] Batool R., Kalsoom A., Akbar I., Arshad N., Jamil N. (2018). Antilisterial effect of Rosa damascena and Nymphaea alba in Mus musculus. BioMed Research International.

[bb0065] Baydar N.G., Baydar H. (2013). Phenolic compounds, antiradical activity and antioxidant capacity of oil-bearing rose (Rosa damascena mill.) extracts. Industrial Crops and Products.

[bb0070] Bikmoradi A., Roshanaei G., Moradkhani S., Fatahi A. (2022). Impact of inhalation aromatherapy with damask rose (Rosa damascena) on stress, anxiety and hemodynamic parameters of patients undergoing coronary angiography: a single blind randomized clinical trial. J Complement Integr Med.

[bb0075] Boskabady M.H., Shafei M.N., Saberi Z., Amini S. (2011). Pharmacological effects of rosa damascena. Iranian Journal of Basic Medical Sciences.

[bb0080] Chávez-Delgado E.L., Jacobo-Velázquez D.A. (2023). Essential oils: Recent advances on their dual role as food preservatives and nutraceuticals against the metabolic syndrome. Foods.

[bb0085] Chochkov R., Denkova R., Denkova Z., Denev P., Vasileva I., Dessev T., Slavov A. (2022). Utilization of industrial Rosa damascena mill. by-products and cocoa pod husks as natural preservatives in muffins. Periodica Polytechnica Chemical Engineering.

[bb0090] Chroho M., Bouymajane A., Oulad El Majdoub Y., Cacciola F., Mondello L., Aazza M., Bouissane L. (2022). Phenolic composition, antioxidant and antibacterial activities of extract from flowers of Rosa damascena from Morocco. Separations.

[bb0095] Contri R.V., Kulkamp-Guerreiro I.C., da Silva S.J., Frank L.A., Pohlmann A.R., Guterres S.S. (2016). Nanoencapsulation of rose-hip oil prevents oil oxidation and allows obtainment of gel and film topical formulations. AAPS PharmSciTech.

[bb0100] Cui W., Xu R., Li X., Yang J., Xu P., Zhang Z., Adiges S. (2024). Research on the supercritical CO2 extraction process of Hetian rose essential oil. Processes.

[bb0105] Dagli R., Avcu M., Metin M., Kiymaz S., Ciftci H. (2019). The effects of aromatherapy using rose oil (Rosa damascena mill.) on preoperative anxiety: A prospective randomized clinical trial. European Journal of Integrative Medicine.

[bb0110] Darvishi Nooshabadi M.A., Sabet J.K., Zahirifar J., Dastbaz A. (2024). Supercritical fluid extraction of damask rose: Optimization, simulation, and economic estimation of process. Korean Journal of Chemical Engineering.

[bb0115] Davoodi I., Rahimi R., Abdollahi M., Farzaei F., Farzaei M.H., Memariani Z., Najafi F. (2017). Promising effect of Rosa damascena extract on high-fat diet-induced nonalcoholic fatty liver. Journal of Traditional and Complementary Medicine.

[bb0120] Díaz-Reinoso B., Moure A., Domínguez H., Parajó J.C. (2006). Supercritical CO2 extraction and purification of compounds with antioxidant activity. Journal of Agricultural and Food Chemistry.

[bb0125] Dinçoğlu A.H., Rugji J. (2021). Use of rose oil in probiotic fermented whey as a functional food. Journal of Food Science and Technology.

[bb0130] Farahnaky A., Gavahian M., Mehraban-Jahromi M. (2010). Design and development of ohmic distillatory device with ability of process monitoring and controlling. Iran patent.

[bb0135] Farnia V., Shirzadifar M., Shakeri J., Rezaei M., Bajoghli H., Holsboer-Trachsler E., Brand S. (2015). Rosa damascena oil improves SSRI-induced sexual dysfunction in male patients suffering from major depressive disorders: results from a double-blind, randomized, and placebo-controlled clinical trial. Neuropsychiatric Disease and Treatment.

[bb0140] Farnia V., Tatari F., Alikhani M., Shakeri J., Taghizadeh M., Karbasizadeh H., Brand S. (2017). Rosa Damascena oil improved sexual function and testosterone in male patients with opium use disorder under methadone maintenance therapy-results from a double-blind, randomized, placebo-controlled clinical trial. Drug and Alcohol Dependence.

[bb0145] Fast D.J., Stern N.P., Chuang J., Li Y., Scholten J.D., Hu C. (2019). Flavanones common to citrus fruits activate the interferon-stimulated response element by stimulating expression of IRF7. Journal of Food Bioactives.

[bb0150] Fathima S.N., Murthy S.V. (2019). Cardioprotective effects to chronic administration of Rosa damascena petals in isoproterenol induced myocardial infarction: biochemical, histopathological and ultrastructural studies. Biomedical and Pharmacology Journal.

[bb0155] Liu W.-Y., Chen L.-Y., Huang Y.-Y., Fu L., Song L.-Y., Wang Y.-Y., Bi Y.-F. (2020). Antioxidation and active constituents analysis of flower residue of Rosa damascena. Chinese Herbal Medicines.

[bb0160] Gateva S., Jovtchev G., Angelova T., Gerasimova T., Dobreva A., Mileva M. (2023). Cytogenetic studies on Genoprotective effect of Rosa damascena mill. Hydrosol in plant and lymphocyte test systems. Life.

[bb0165] Getachew M., Awoke S., Melaku Y., Gashu M., Gizachew Z. (2019). Formulation of substantial natural flavors from plant materials for food and beverage industries. Journal of Food Processing & Technology.

[bb0170] Ghavam M., Afzali A., Manca M.L. (2021). Chemotype of damask rose with oleic acid (9 octadecenoic acid) and its antimicrobial effectiveness. Scientific Reports.

[bb0175] Ghorbani Rami M.S., Nasiri M., Aghili Nasab M.S., Jafari Z., Torkaman M., Feizi S., Asadi M. (2021). Effect of Rosa damascena on improvement of adults’ sleep quality: a systematic review and meta-analysis of randomized controlled trials. Sleep Medicine.

[bb0180] Giannakourou M.C., Tsironi T., Thanou I., Tsagri A.M., Katsavou E., Lougovois V., Sinanoglou V.J. (2019). Shelf life extension and improvement of the nutritional value of fish fillets through osmotic treatment based on the sustainable use of Rosa damascena distillation by-products. Foods.

[bb0185] Gunawardana S.L.A., Jayasuriya W. (2019). Medicinally important herbal flowers in Sri Lanka. Evidence-based Complementary and Alternative Medicine.

[bb0190] Guo C., Zhang J., Liu C., Bian Y., Shan Q. (2022). Extracting rose essential oil from rose slag with ionic liquid. Biomass Conversion and Biorefinery.

[bb0195] Hadian, Z., M. Kamalabadi, Y. Phimolsiripol, B. Balasubramanian, J. Manuel Lorenzo Rodriguez and A. Mousavi Khaneghah (2023). "Preparation, characterization, and antioxidant activity of β-cyclodextrin nanoparticles loaded Rosa damascena essential oil for application in beverage." Food Chemistry 403: 134410. 10.1016/j.foodchem.2022.134410 36183469

[bb0200] Halawani E.M. (2014). Antimicrobial activity of Rosa damascena petals extracts and chemical composition by gas chromatography-mass spectrometry (GC/MS) analysis. African Journal of Microbiology Research.

[bb0205] Hamdamian S., Nazarpour S., Simbar M., Hajian S., Mojab F., Talebi A. (2018). Effects of aromatherapy with Rosa damascena on nulliparous women’s pain and anxiety of labor during first stage of labor. Journal of Integrative Medicine.

[bb0210] He A., Putra N.R. (2025). Rose essential oils: Current trends, mapping of extraction techniques, chemical analysis, therapeutic applications, and by-product valorization. The Canadian Journal of Chemical Engineering.

[bb0215] He Z., Wang H., Wang W., Shen X., Yu C., Yue H., Yao L. (2024). The volatile and sensorial profiles of processed damask rose (Rosa damascena mill.) products cultivated in China under different process treatments. Industrial Crops and Products.

[bb0220] Hosseini F.Z., Behmanesh F., Mirabi P., Memariani Z., Nikpour M., Omidvar S., Aghamohammadi A. (2024). Aromatherapy with Rosa damascena mill. to relieve the symptoms of postpartum depression and sleep quality in primiparous women: A randomised controlled trial. Journal of Herbal Medicine.

[bb0225] Huang F.-C., Horváth G., Molnár P., Turcsi E., Deli J., Schrader J., Sandmann G., Schmidt H., Schwab W. (2009). Substrate promiscuity of RdCCD1, a carotenoid cleavage oxygenase from Rosa damascena. Phytochemistry.

[bb0230] Imanieh M.H., Honar N., Mohagheghzadeh A., Haghighat M., Dehghani S.M., Mosleh G., Avazpour A. (2022). Rosa damascena together with brown sugar mitigate functional constipation in children over 12 months old: A double-blind randomized controlled trial. Journal of Ethnopharmacology.

[bb0235] Jiao Y., Liu T., Zhou S., Xu Y. (2024). Evaluation of antioxidant and intelligent films for pork packaging and spoilage monitoring based on maize starch, carrageenan gum, and Rosa chinensis flower extracts. Journal of Food Measurement and Characterization.

[bb0240] Kanatt S.R. (2021). Active/smart carboxymethyl cellulose-polyvinyl alcohol composite films containing rose petal extract for fish packaging. International Journal of Food Science & Technology.

[bb0245] Kang S., Wang H., Xia L., Chen M., Li L., Cheng J., Li X., Jiang S. (2020). Colorimetric film based on polyvinyl alcohol/okra mucilage polysaccharide incorporated with rose anthocyanins for shrimp freshness monitoring. Carbohydrate Polymers.

[bb0250] Kara, N., S. Erbaş and H. Baydar (2017). "The effect of seawater used for hydrodistillation on essential oil yield and composition of oil-bearing Rose (Rosa damascena Mill.)." International Journal of Secondary Metabolite **4**(3, Special Issue 2): 423–428.

[bb0255] Karami A., Khosh-Khui M., Salehi H., Saharkhiz M.J. (2012). Correlation between anthocyanin and essential oil content of damask rose (Rosa damascena mill.). Journal of Medicinal plants and By-product.

[bb0260] Kashani L.M.-T., Memarzadeh M.R., Hatami A., Shirzad M., Ahmadian-Attari M.M. (2016). Comparison of two different traditional methods of rose oil preparation in terms of physicochemical factors. Traditional and Integrative Medicine.

[bb0265] Katekar V.P., Rao A.B., Sardeshpande V.R. (2022). Review of the rose essential oil extraction by hydrodistillation: An investigation for the optimum operating condition for maximum yield. Sustainable Chemistry and Pharmacy.

[bb0270] Kayahan S., Gülbağ F., Kaya Y., Altunkanat H. (2024). Determination of phenolic, flavonoid content and antioxidant activity of oil rose products. Horticultural Studies.

[bb0275] Khodadadi F., Nikzad M., Hamedi S. (2024). Lignin nanoparticles as a promising nanomaterial for encapsulation of rose damascene essential oil: Physicochemical, structural, antimicrobial and in-vitro release properties. Colloids and Surfaces A: Physicochemical and Engineering Aspects.

[bb0280] Kiani H.S., Noudehi M.S., Shokrpour M., Zargar M., Naghavi M.R. (2024). Investigation of genes involved in scent and color production in Rosa damascena mill. Scientific Reports.

[bb0285] Kolev N.D. (2022). Natural antioxidants–an alternative for reduction of nitrites in cooked meat products. Food Science And Applied Biotechnology.

[bb0290] Konteles S.J., Stavropoulou N.A., Thanou I.V., Mouka E., Kousiaris V., Stoforos G.N., Giannakourou M.C. (2023). Enriching cured meat products with bioactive compounds recovered from Rosa damascena and Rosmarinus officinalis L. distillation by-products: The pursuit of natural antimicrobials to reduce the use of nitrites. Applied Sciences.

[bb0295] Koraqi H., Aydar A.Y., Khalid W., Ercisli S., Rustagi S., Ramniwas S., Pandiselvam R. (2024). Ultrasound-assisted extraction with natural deep eutectic solvent for phenolic compounds recovery from Rosa damascene mill.: Experimental design optimization using central composite design. Microchemical Journal.

[bb0300] Kord Heydari M., Assadpour E., Jafari S.M., Javadian H. (2021). Encapsulation of rose essential oil using whey protein concentrate-pectin nanocomplexes: Optimization of the effective parameters. Food Chemistry.

[bb0305] Kumar A., Kaur A., Tomer V., Gupta K., Kaur K. (2021). Effect of rose syrup and Marigold powder on the physicochemical, phytochemical, sensorial and storage properties of Nutricereals and Milk-based functional beverage. Journal of the American College of Nutrition.

[bb0310] Li Y., Wang J.-H., Wang E.-C., Tang Z.-S., Han Y., Luo X.-E., Zeng X.-A., Woo M.-W., Han Z. (2023). The microstructure and thermal properties of pulsed electric field pretreated oxidized starch. International Journal of Biological Macromolecules.

[bb0315] Liu Y., Liu R., Shi J., Zhang R., Tang H., Xie C., Wang F., Han J., Jiang L. (2023). Chitosan/esterified chitin nanofibers nanocomposite films incorporated with rose essential oil: Structure, physicochemical characterization, antioxidant and antibacterial properties. Food Chemistry: X.

[bb0320] Mahdood B., Imani B., Khazaei S. (2022). Effects of inhalation aromatherapy with Rosa damascena (damask rose) on the state anxiety and sleep quality of operating room personnel during the COVID-19 pandemic: A randomized controlled trial. Journal of Perianesthesia Nursing.

[bb0325] Mahmood N., Piacente S., Pizza C., Burke A., Khan A.I., Hay A.J. (1996). The anti-HIV activity and mechanisms of action of pure compounds isolated fromRosa damascena. Biochemical and Biophysical Research Communications.

[bb0330] Manouchehri R., Saharkhiz M.J., Karami A., Niakousari M. (2018). Extraction of essential oils from damask rose using green and conventional techniques: Microwave and ohmic assisted hydrodistillation versus hydrodistillation. Sustainable Chemistry and Pharmacy.

[bb0335] Maqsood-ul-Haque S., Shariffuddin N.S.M. (2022). Development of biodegradable food packaging incorporated with pigment of rose and red cabbage. International Journal of Integrated Engineering.

[bb0340] Mawarni E., Ginting C.N., Chiuman L., Girsang E., Handayani R.A.S., Widowati W. (2020). Antioxidant and elastase inhibitor potential of petals and receptacle of rose flower (Rosa damascena). Pharm. Sci. Res.

[bb0345] Memariani, Z., G. Amin, G. Moghaddam and M. Hajimahmoodi (2015). "Comparative analysis of phenolic compounds in two samples of Rosa damascena by HPLC.".

[bb0350] Meng J.-R., Liu J., Fu L., Shu T., Yang L., Zhang X., Bai L.-P. (2023). Anti-entry activity of natural flavonoids against SARS-CoV-2 by targeting spike RBD. Viruses.

[bb0355] Mileva M., Ilieva Y., Jovtchev G., Gateva S., Zaharieva M.M., Georgieva A., Vilhelmova-Ilieva N. (2021). Rose flowers—A delicate perfume or a natural healer?. Biomolecules.

[bb0360] Mohammadi B., Shekaari H., Zafarani-Moattar M.T. (2021). Separation and encapsulation of Persian red rose oil by eutectic compounds. Microchemical Journal.

[bb0365] Mokhtari R., Ajorpaz N.M., Abdi K., Golitaleb M. (2023). The effects of Rosa damascene aromatherapy on anxiety and sleep quality in burn patients: A randomized clinical trial. Burns.

[bb0370] Monfared B.P., Ahari H., Moradi S., Sahraei F. (2024). Investigation of damask rose extract nanoemulsions as a shelf-life extender for refrigerated beluga sturgeon fillets. Journal of Food Measurement and Characterization.

[bb0375] Mustafa A., El-Kashef D.H., Abdelwahab M.F., Gomaa A.A.R., Mustafa M., Abdel-Wahab N.M., Ibrahim A.H. (2023). Investigation of antiviral effects of essential oils. Essential Oils: Extraction Methods and Applications.

[bb0380] Nasery, M., M. K. Hassanzadeh, Z. T. Najaran and S. A. Emami (2016). Chapter 75 - Rose (Rosa×damascena Mill.) Essential Oils. Essential Oils in Food Preservation, Flavor and Safety. V. R. Preedy. San Diego, Academic Press**:** 659–665.

[bb0385] Niazi M., Hashempur M.H., Taghizadeh M., Heydari M., Shariat A. (2017). Efficacy of topical rose (Rosa damascena mill.) oil for migraine headache: A randomized double-blinded placebo-controlled cross-over trial. Complementary Therapies in Medicine.

[bb0390] Niazi P., Hejran A.B., Alimyar O. (2025). Assessment of antibacterial and antifungal activities of ethanolic flower extracts from Rosa damascena against pathogenic Micro-organisms. Black Sea Journal of Agriculture.

[bb0395] Omidi M., Khandan-Mirkohi A., Kafi M., Rasouli O., Shaghaghi A., Kiani M., Zamani Z. (2022). Comparative study of phytochemical profiles and morphological properties of some damask roses from Iran. Chemical and Biological Technologies in Agriculture.

[bb0400] Önder D. (2023). Variation in antioxidant capacity, antioxidant activity and mineral composition during flower development of oil-bearing rose (Rosa damascena mill.). Scientific Reports.

[bb0405] Osama, M., R. Ikram, C. R. Wei, R. Saleem, G. R. Bhurgri, F. J. Siyal and W. Abbas (2022). "Biochemical Screening Of Unani Herbal Product “Arq-E-Gulab” For Its Chronic Effects On Serum Creatinine Levels." NVEO-NATURAL VOLATILES & ESSENTIAL OILS Journal| NVEO: 13607–13612.

[bb0410] Patrascu M., Radoiu M. (2016). Rose essential oil extraction from fresh petals using synergetic microwave & ultrasound energy: Chemical composition and antioxidant activity assessment. J. Chem. Chem. Eng.

[bb0415] Pensamiento-Niño C.A., Castañeda-Ovando A., Añorve-Morga J., Hernández-Fuentes A.D., Aguilar-Arteaga K., Ojeda-Ramírez D. (2024). Edible flowers and their relationship with human health: Biological activities. Food Reviews International.

[bb0420] Pereira A.M., Cruz R.R.P., Gadelha T.M., da Silva Á.G.F., da Costa F.B., Ribeiro W.S. (2020). Edible flowers: beauty, health and nutrition. Research, Society and Development.

[bb0425] Pires T.C.S.P., Dias M.I., Barros L., Barreira J.C.M., Santos-Buelga C., Ferreira I.C.F.R. (2018). Incorporation of natural colorants obtained from edible flowers in yogurts. LWT.

[bb0430] Pise V.H., Harlalka R., Thorat B.N. (2023).

[bb0435] Prosvetova A., Samsonenko S., Poyedynok N. (2023). Mechanisms of antiviral activity of flavonoids. Biotechnologia acta.

[bb0440] Qiu L., Zhang M., Adhikari B., Chang L. (2022). Microencapsulation of rose essential oil in mung bean protein isolate-apricot peel pectin complex coacervates and characterization of microcapsules. Food Hydrocolloids.

[bb0445] Qiu L., Zhang M., Adhikari B., Chang L. (2023). Microencapsulation of rose essential oil using Perilla protein isolate-sodium alginate complex coacervates and application of microcapsules to preserve ground beef. Food and Bioprocess Technology.

[bb0450] Qiu L., Zhang M., Huang M., Chitrakar B., Chang L. (2023). Fabrication of sodium alginate/apricot peel pectin films incorporated with rose anthocyanin-rich extract for monitoring grass carp (Ctenopharyngodon idellus) freshness. Food Packaging and Shelf Life.

[bb0455] Rajabi-Moghadam M., Mahdavi B., Habibi A., Mahdi M. (2025). Phytochemical composition of Rosa foetida essential oil and evaluation of antioxidant and anti-oral squamous Cancer activities. Jundishapur Journal of Natural Pharmaceutical Products.

[bb0460] Rasooli T., Nasiri M., Kargarzadeh Aliabadi Z., Rajabi M.R., Feizi S., Torkaman M., Abbasi M. (2021). Rosa Damascena mill for treating adults’ anxiety, depression, and stress: A systematic review and dose-response meta-analysis of randomized controlled trials. Phytotherapy Research.

[bb0465] Rasouli O., Ahmadi N., Rashidi Monfared S., Sefidkon F. (2018). Physiological, phytochemicals and molecular analysis of color and scent of different landraces of Rosa damascena during flower development stages. Scientia Horticulturae.

[bb0470] S, E.-S., Abdel-Hameed, S. A. Bazaid and M. S. Salman (2013). "Characterization of the phytochemical constituents of Taif rose and its antioxidant and anticancer activities." BioMed Research International 2013(1): 345465. 10.1155/2013/345465 PMC3825121 24282813

[bb0475] Sadeghi Mansoorkhani H., Alamdari M., Hasanzadeh S., Jokar S., Panahi Kokhdan E., Malekzadeh J., Abassi R. (2021). Effect of Rosa damascene mill. Hydroalcoholic extract on the treatment of chronic idiopathic constipation in adults. Journal of Clinical Care and Skills.

[bb0480] Saffari I., Motallebi Moghanjoghi A., Sharafati Chaleshtori R., Ataee M., Khaledi A. (2023). Nanoemulsification of rose (Rosa damascena) essential oil: Characterization, anti-Salmonella, in vitro cytotoxicity to Cancer cells, and advantages in sheep meat application. Journal of Food Quality.

[bb0485] Sani I.K., Mehrnoosh F., Rasul N.H., Hassani B., Mohammadi H., Gholizadeh H., Sani M.A. (2024). “pulsed electric field-assisted extraction of natural colorants; principles and applications.” food. Bioscience.

[bb0490] Sani M.A., Priyadarshi R., Zhang W., Khezerlou A., Rhim J.-W. (2024). Innovative application of laccase enzyme in food packaging. Trends in Food Science & Technology.

[bb0495] Shabbir, F., M. A. Hanif, M. A. Ayub, M. I. Jilani and S. Rahman (2020). Chapter 17 - Damask Rose. Medicinal Plants of South Asia. M. A. Hanif, H. Nawaz, M. M. Khan and H. J. Byrne, Elsevier**:** 217–230.

[bb0500] Shavisi N., Shahbazi Y. (2022). Chitosan-gum Arabic nanofiber mats encapsulated with pH-sensitive Rosa damascena anthocyanins for freshness monitoring of chicken fillets. Food Packaging and Shelf Life.

[bb0505] Shi Y., Wu Y.-W., Jin H.-F., Jiao Y.-H., Cao J., Ye L.-H. (2024). In situ ionic liquid-assisted mechanochemical extraction and aqueous two-phase system enrichment of hydrophobic flavonoids from rose. Industrial Crops and Products.

[bb0510] Shokrzadeh M., Habibi E., Modanloo M. (2017). Cytotoxic and genotoxic studies of essential oil from Rosa damascene mill., Kashan, Iran. Medicinski Glasnik.

[bb0515] Thakur D., Kumar Y., Sharanagat V.S., Srivastava T., Saxena D.C. (2023). Development of pH-sensitive films based on buckwheat starch, critic acid and rose petal extract for active food packaging. Sustainable Chemistry and Pharmacy.

[bb0520] Torusdağ G.B., Bakkalbaşı E. (2022). Determination of some physicochemical properties and anthocyanin extraction conditions of Rosa damascena mill. Journal of Food Processing and Preservation.

[bb0525] Trendafilova A., Staleva P., Petkova Z., Ivanova V., Evstatieva Y., Nikolova D., Simova S. (2023). Phytochemical profile, antioxidant potential, antimicrobial activity, and cytotoxicity of dry extract from Rosa damascena mill. Molecules.

[bb0530] Venkatesha K.T., Gupta A., Rai A.N., Jambhulkar S.J., Bisht R., Padalia R.C. (2022). Recent developments, challenges, and opportunities in genetic improvement of essential oil-bearing rose (Rosa damascena): A review. Industrial Crops and Products.

[bb0535] Verešová A., Vukic M.D., Vukovic N.L., Terentjeva M., Ban Z., Li L., Kačániová M. (2024). Chemical composition, biological activity, and application of Rosa damascena essential oil as an antimicrobial agent in minimally processed eggplant inoculated with Salmonella enterica. Foods.

[bb0540] Vilhelmova-Ilieva N., Dobreva A., Doynovska R., Krastev D., Mileva M. (2021). Antiviral activity of Rosa damascena mill. and Rosa alba L. essential oils against the multiplication of herpes simplex virus type 1 strains sensitive and resistant to acyclovir. Biology.

[bb0545] Villa, C., F. S. Robustelli Della Cuna, E. Russo, M. F. Ibrahim, E. Grignani and S. Preda (2022). "Microwave-assisted and conventional extractions of volatile compounds from Rosa x damascena mill. Fresh petals for cosmetic applications." Molecules 27(12): 3963. 10.3390/molecules27123963 PMC9228154 35745086

[bb0550] Wagh R.V., Riahi Z., Kim J.T., Rhim J.-W. (2024). Carrageenan-based functional films hybridized with carbon dots and anthocyanins from rose petals for smart food packaging applications. International Journal of Biological Macromolecules.

[bb0555] Wahyuningsih S., Wulandari L., Wartono M., Munawaroh H., Ramelan A. (2017).

[bb0560] Wan H., Yu C., Han Y., Guo X., Luo L., Pan H., Zhang Q. (2019). “determination of flavonoids and carotenoids and their contributions to various colors of rose cultivars (Rosa spp.).” Frontiers. Plant Science.

[bb0565] Wang C., Lu Y., An X., Wang Y., Wang N., Song Y., Hu N., Ren M. (2024). Preparation, characterization, and application of pH-responsive biodegradable intelligent indicator film based on rose anthocyanins. LWT.

[bb0570] Wang H., Fan Y., Yang Y., Zhang H., Li M., Sun P., Zhang X., Xue Z., Jin W. (2023). Classification of rose petal colors based on optical spectrum and pigment content analyses. Horticulture, Environment, and Biotechnology.

[bb0575] Wang Y., Zhang J., Zhang L. (2022). An active and pH-responsive film developed by sodium carboxymethyl cellulose/polyvinyl alcohol doped with rose anthocyanin extracts. Food Chemistry.

[bb0580] Wen Z., You X., Jiang L., Liu B., Zheng Z., Pu Y., Cheng B. (2011). Liposomal incorporation of rose essential oil by a supercritical process. Flavour and Fragrance Journal.

[bb0585] Wu L., Zhang C., Long Y., Chen Q., Zhang W., Liu G. (2022). Food additives: From functions to analytical methods. Critical Reviews in Food Science and Nutrition.

[bb0590] Wu W., Zheng L., Yu J., Liu L., Goksen G., Shao P. (2023). High-sensitivity intelligent packaging films harnessing rose anthocyanins and hydrophilic silica aerogel for visual food freshness monitoring. Food Quality and Safety.

[bb0595] Xiao Z., Kang Y., Hou W., Niu Y., Kou X. (2019). Microcapsules based on octenyl succinic anhydride (OSA)-modified starch and maltodextrins changing the composition and release property of rose essential oil. International Journal of Biological Macromolecules.

[bb0600] Xue F., Li C., Adhikari B. (2024). Physicochemical properties of active films of rose essential oil produced using soy protein isolate-polyphenol conjugates for cherry tomato preservation. Food Chemistry.

[bb0605] Yajun Z., Changmei X., Susu Z., Guangming Y., Ling Z., Shujie W. (2017). Effects of high intensity pulsed electric fields on yield and chemical composition of rose essential oil. International Journal of Agricultural and Biological Engineering.

[bb0610] Yang Y., Yu X., Zhu Y., Zeng Y., Fang C., Liu Y., Hu S., Ge Y., Jiang W. (2022). Preparation and application of a colorimetric film based on sodium alginate/sodium carboxymethyl cellulose incorporated with rose anthocyanins. Food Chemistry.

[bb0615] Yari E., Sari S., Kelidari H., Asare-Addo K., Nokhodchi A. (2024). Effect of Rosa damascena essential oil loaded in nanostructured lipid carriers on the proliferation of human breast Cancer cell line MDA-MB-231 in comparison with cisplatin. Journal of Pharmaceutical Innovation.

[bb0620] Yazdanfar N., Mohamadi S., Zienali T., Sadighara P. (2022). Quantification of methanol, ethanol, and essential oil contents of commonly used Brands of Rosewater (Rosa Damascena) in Iran. Journal of Nutrition and Food Security.

[bb0625] Zahedi-Amiri Z., Taravati A., Hejazian L.B. (2019). Protective effect of Rosa damascena against Aluminum chloride-induced oxidative stress. Biological Trace Element Research.

[bb0630] Zakaryan H., Arabyan E., Oo A., Zandi K. (2017). Flavonoids: promising natural compounds against viral infections. Archives of Virology.

[bb0635] Zang E., Jiang L., Cui H., Li X., Yan Y., Liu Q., Chen Z., Li M. (2023). Only plant-based food additives: An overview on application, safety, and key challenges in the food industry. Food Reviews International.

[bb0640] Zhang W., Jiang H., Rhim J.-W., Cao J., Jiang W. (2022). Effective strategies of sustained release and retention enhancement of essential oils in active food packaging films/coatings. Food Chemistry.

[bb0645] Zheng L., Liu L., Yu J., Farag M.A., Shao P. (2023). Intelligent starch/chitosan-based film incorporated by anthocyanin-encapsulated amylopectin nanoparticles with high stability for food freshness monitoring. Food Control.

